# Epigenome-wide association study of total serum immunoglobulin E in children: a life course approach

**DOI:** 10.1186/s13148-018-0488-x

**Published:** 2018-04-17

**Authors:** Cheng Peng, Andres Cardenas, Sheryl L. Rifas-Shiman, Marie-France Hivert, Diane R. Gold, Thomas A. Platts-Mills, Xihong Lin, Emily Oken, Andrea A. Baccarelli, Augusto A. Litonjua, Dawn L. DeMeo

**Affiliations:** 1 Channing Division of Network Medicine and the Division of Pulmonary and Critical Care Medicine, Department of Medicine, Brigham and Women’s Hospital, Harvard Medical School, 181 Longwood Avenue, 4th Floor, Boston, MA 02115 USA; 2 Division of Chronic Disease Research Across the Lifecourse, Department of Population Medicine, Harvard Medical School and Harvard Pilgrim Health Care Institute, Boston, MA USA; 3 Diabetes Unit, Massachusetts General Hospital, Boston, MA USA; 4 Department of Environmental Health, Harvard T. H. Chan School of Public Health, Boston, MA USA; 5 Division of Allergy and Clinical Immunology, University of Virginia School of Medicine, Charlottesville, VA USA; 6 Department of Biostatistics, Harvard T. H. Chan School of Public Health, Boston, MA USA; 7 Department of Environmental Health Sciences, Columbia University Mailman School of Public Health, New York, NY USA

**Keywords:** Epigenome-wide association studies, Total serum IgE, Life course analysis

## Abstract

**Background:**

IgE-mediated sensitization may be epigenetically programmed in utero, but early childhood environment may further alter complex traits and disease phenotypes through epigenetic plasticity. However, the epigenomic footprint underpinning IgE-mediated type-I hypersensitivity has not been well-understood, especially under a longitudinal early-childhood life-course framework.

**Methods:**

We used epigenome-wide DNA methylation (IlluminaHumanMethylation450 BeadChip) in cord blood and mid-childhood peripheral blood to investigate pre- and post-natal methylation marks associated with mid-childhood (age 6.7–10.2) total serum IgE levels in 217 mother-child pairs in Project Viva—a prospective longitudinal pre-birth cohort in eastern Massachusetts, USA. We identified methylation sites associated with IgE using covariate-adjusted robust linear regressions.

**Results:**

Nineteen methylation marks in cord blood were associated with IgE in mid-childhood (FDR < 0.05) in genes implicated in cell signaling, growth, and development. Among these, two methylation sites (*C7orf50*, *ZAR1*) remained robust after the adjustment for the change in DNA methylation from birth to mid-childhood (FDR < 0.05). An analysis of the change in methylation between cord blood and mid-childhood DNA (Δ-DNAm) identified 395 methylation marks in 272 genes associated with mid-childhood IgE (FDR < 0.05), with multiple sites located within *ACOT7* (4 sites), *EPX* (5 sites), *EVL* (3 sites), *KSR1* (4 sites), *ZFPM1* (3 sites), and *ZNF862* (3 sites). Several of these methylation loci were previously associated with asthma (*ADAM19*, *EPX*, *IL4*, *IL5RA*, and *PRG2*).

**Conclusion:**

This study identified fetally programmed and mid-childhood methylation signals associated with mid-childhood IgE. Epigenetic priming during fetal development and early childhood likely plays an important role in IgE-mediated type-I hypersensitivity.

**Electronic supplementary material:**

The online version of this article (10.1186/s13148-018-0488-x) contains supplementary material, which is available to authorized users.

## Background

Immunoglobulin E (IgE)—a central mediator for type I hypersensitivity—contributes to the pathogenesis of a wide range of childhood-onset allergic diseases, including asthma, allergic rhinitis, atopic dermatitis, and food allergy [1–4]. IgE-mediated allergic sensitization has its roots very early in life and is likely impacted by the in utero environment [5, 6]. The manifestation of IgE-mediated responses often involves childhood re-exposures to antigens in sensitized individuals, with subsequent IgE-dependent release of inflammatory mediators that give rise to allergic symptoms [3, 4].

To date, the most commonly used therapies for IgE-mediated allergic responses in children focus on acute and chronic symptom relief [7]; no treatment targets the natural history of allergy pathogenesis across the life course. Understanding the molecular origins and mechanisms of IgE-mediated allergic responses, and the plasticity of the associated molecular markers, would inform more effective screening, prevention, and treatment strategies.

Epigenetic regulatory elements, such as DNA methylation marks, undergo dynamic reprogramming during embryogenesis [8, 9]. The establishment of the human methylome during fetal development coincides with early immune development relevant to IgE-mediated allergic sensitization and makes DNA methylation in cord blood a potential early molecular marker of IgE-mediated disease onset. DNA methylation patterns may vary after birth to reflect the complex interplay between genetics, development and maturation, and environmental exposures. Therefore, plasticity of DNA methylation from birth may portend the development and progression of diseases that were driven by both genetic endowment and the environment, such as IgE-mediated allergic phenotypes during childhood.

Previous studies have reported differential DNA methylation in peripheral blood associated with total serum IgE measures [10–13]. However, these studies were either cross-sectional or case-control studies; the lack of prospective nature of these studies impedes the identification of epigenetic marks that predict development of allergy. Further, most of these studies were conducted in later childhood and adulthood. Since IgE-mediated allergic responses may have a fetal origin, these studies may not capture alterations during critical developmental windows, e.g., the period of fetal immune development during which IgE-dependent predisposition may originate.

**Fig. 1:**
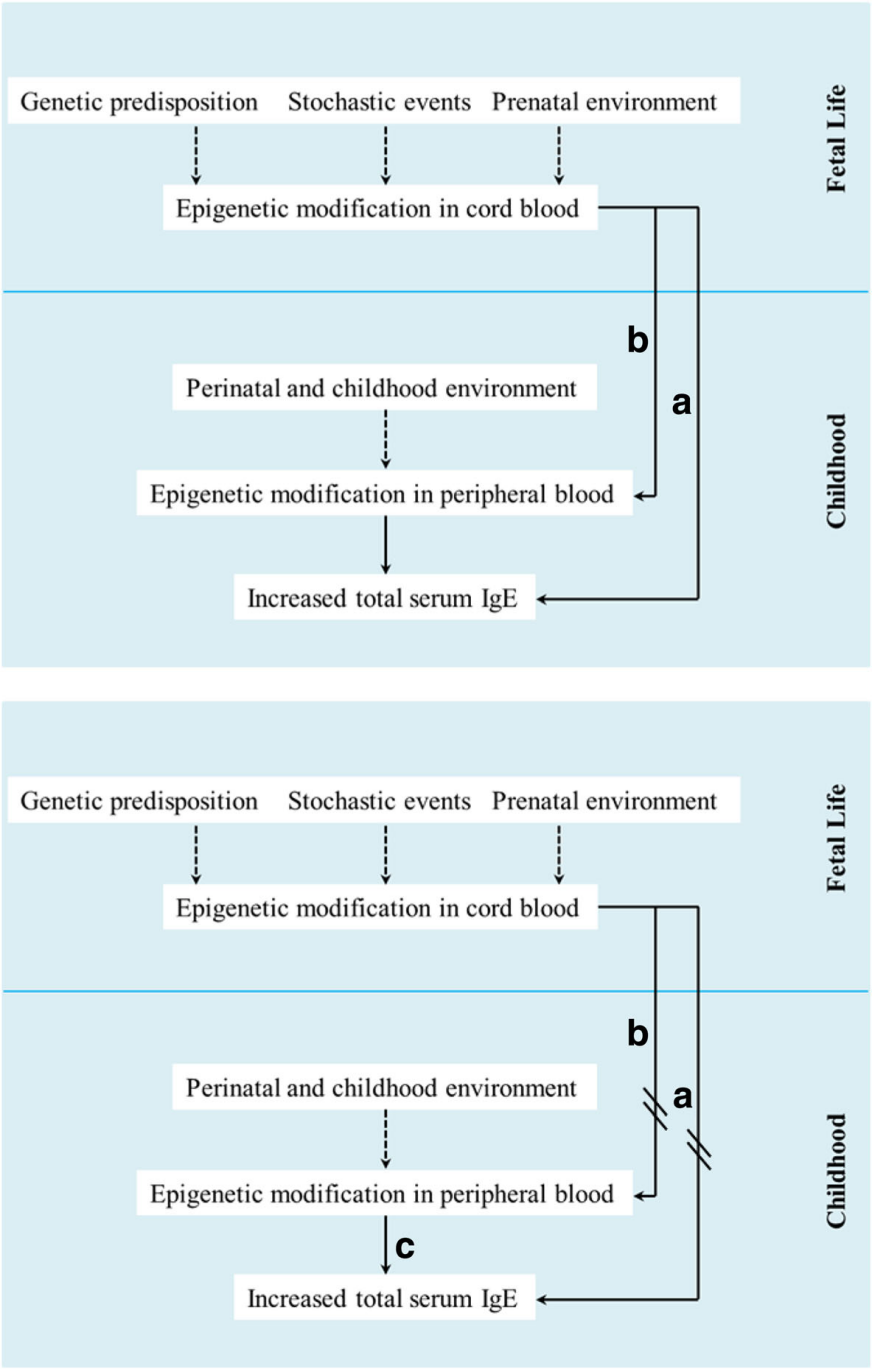
Conceptural model of the lifecourse framework. Path (a): epigenetic modifications established at birth may be associated with IgE levels in childhood independent of factors operating postnatally; path (b): epigenetic marks established at birth may be further influenced by postnatal experiences and exposures; and path (c): postnatal experiences and exposures may be associated with IgE levels independent of prenatal factors

To better understand the molecular origins and progression of IgE-mediated responses, we sought to conceptualize the association between epigenome-wide differential methylation in DNA from blood cells and serum IgE measures using a life course framework [14] (Fig. 1). Specifically, we used methylation data from DNA from blood cells at two distinct time points—birth and mid-childhood (mean age = 7.8 years)—to quantify and dissect the influence of prenatal and childhood factors on IgE. We hypothesize that (1) epigenetic marks in cord blood DNA reflecting the prenatal environment serve as early markers of IgE-mediated allergic response susceptibility in childhood, (2) some epigenetic marks established at birth may be associated with IgE levels in childhood independent of factors operating postnatally, and (3) childhood environmental influences—captured by changes in DNA methylation levels between cord blood and mid-childhood peripheral blood—reflect the progression and manifestation of IgE-mediated allergic responses.

## Methods

### Study population

Our study population is drawn from Project Viva—a prospective longitudinal pre-birth cohort from Massachusetts, USA. All participants were recruited at their initial obstetric visit at Atrius Harvard Vanguard Medical Associates from 1999 to 2002. Eligible participants were those with single gestation, gestational age < 22 weeks at enrollment, able to answer questions in English, and intended to stay in the study region before delivery. A detailed cohort profile has been published previously [13]. At enrollment, mothers provided information on age at enrollment, smoking habits (never smoker/former smoker/smoked during pregnancy), education level, and familial atopy history of asthma, eczema, or hay fever. We collected date of birth and sex from hospital records and calculated gestational age at birth as previously described [13]. Trained research assistants conducted in-person research visits with mother-child pairs in the hospital following delivery, in early childhood (mean age = 3.3 years), and mid-childhood (mean age = 7.8 years). Mothers reported child race/ethnicity in early childhood.

**Fig. 2:**
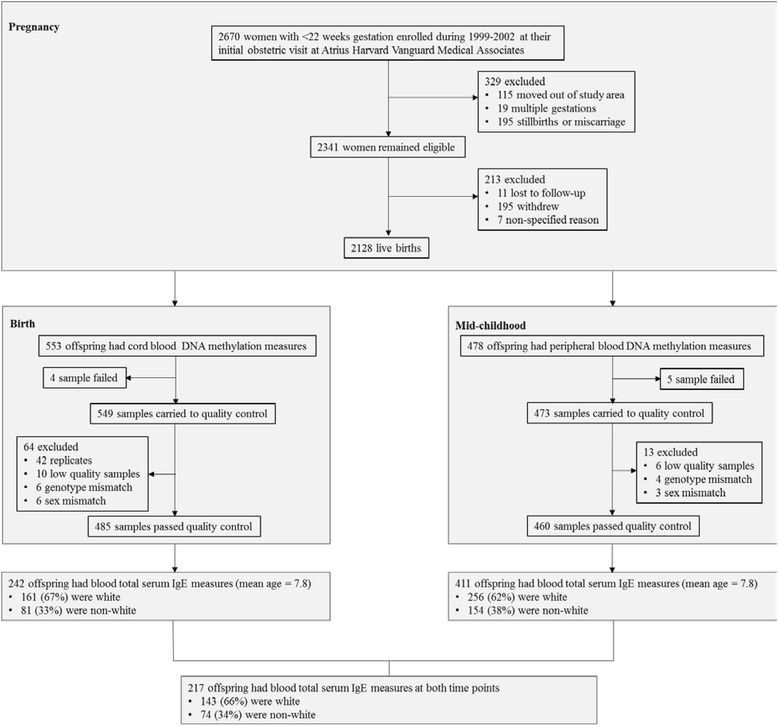
Study flow chart

Of the original 2128 mother-child pairs in the cohort, 616 children had mid-childhood plasma total IgE measures. Among those, 242 children had cord blood DNA methylation measurements at birth, 68 had peripheral blood DNA methylation measurement at early childhood, and 411 had peripheral blood DNA methylation measurements at mid-childhood. Two hundred and seventeen children had DNA methylation measurements at both birth and mid-childhood, while 56 had DNA methylation measurements at all three time points (at birth, early childhood, and mid-childhood) (Fig. 2).

### DNA methylation measures

Trained personnel collected blood samples at birth (umbilical cord blood) and early and mid-childhood (peripheral blood). DNA was extracted using the Qiagen Puregene Kit (Valencia, CA) and bisulfite converted using the EZ DNA Methylation-Gold Kit (Zymo Research, Irvine, CA). Samples were allocated to chips and plates using a two-stage randomization algorithm and analyzed with Infinium HumanMethylation450 BeadChip (Illumina, San Diego, CA), which includes ~ 485,000 CpG sites at a single nucleotide resolution. In the quality control step, we removed technical replicates, samples with low quality, genotype mismatch, and sex mismatch. At the probe level, low-quality probes with detection *P* values > 0.05, probes on sex chromosomes, SNP-associated probes, non-CpG probes, and non-specific and previously identified cross-reactive probes were excluded [15]. We further removed probes within 10 base pairs of a known SNP (UCSC Human Feb. 2009 [GRCh37/hg19] Assembly) with a minor allele frequency (MAF) ≥ 1%. After the quality control, a total of 343,208 probes were included for subsequent analysis. We used the normal-exponential out-of-band (“noob”) method for background correction and dye bias adjustment [16] and normalized our sample with Beta Mixture Quantile Dilation (BMIQ) [17]. To control for technical variability across sample plates, we applied Combat [18]. We report the percent of methylation for each CpG sites as *β* values, calculated as the signal intensity of methylated cytosines over the signal intensity of methylated and un-methylated cytosines at the 5C position [*β* value = M/(M + U)]. In order to control for cell-type heterogeneity, we applied ReFACTor—a reference-free method based on principle component analysis (PCA) with low rank approximation [19]. We chose this method because ReFACTor components correlate well with measured eosinophil and neutrophil counts [19]. Eosinophils and neutrophils are central effector immune cells that often associate with allergic outcomes. By adequately adjusting for eosinophil and neutrophil counts, we aimed to reduce the influence of changes in eosinophil and neutrophil cellular composition. We computed the first five ReFACTor components in cord blood and the first five ReFACTor components in mid-childhood peripheral blood, and used them in subsequent analysis for the adjustment of cell type composition.

### Total serum IgE measures

Total serum IgE concentrations were measured in mid-childhood blood using ImmunoCAP (Phadia, Uppsala, Sweden)—a widely used in vitro sandwich immunoassay to quantitatively measure circulating IgE in serum samples.

### Statistical analyses

#### Cord blood DNA methylation and childhood total serum IgE

We evaluated the association of DNA methylation at each individual CpG site in cord blood with total serum IgE measured in mid-childhood using robust linear regression. To remove the influence of extreme methylation outliers, we restricted the methylation values between [25th percentile − 3*IQR] and [75th percentile + 3*IQR], where IQR represents interquantile range. Total serum IgE was natural log-transformed, and model estimates are expressed as the change in the log total serum IgE per 1% increase in methylation values. Regression models were adjusted for the following covariates selected a priori: maternal [age at enrollment (continuous), smoking status (never, former, or during pregnancy), college graduate (yes/no), maternal atopy history (yes/no)], child [sex (female/male), race/ethnicity (white/black/others), gestational age at birth (continuous), age at blood drawn (continuous)], and the first five ReFACTor components computed from cord blood to capture cellular heterogeneity [19]. A methylation site was considered to be epigenome-wide significant if it reached the false discovery rate (FDR)-corrected *p* value of less than 0.05 based on the method of Benjamini and Hochberg [20]. Additional methylation signals, which were marginally significant (FDR < 0.1), are included in the supplement. The main regression model took the general form:

$$ \log \left(\mathrm{IgE}\right)={\upalpha}_0+{\upalpha}_1\mathrm{CpG}{\_}_{\mathrm{cbi}}+{\upalpha}_2{\mathrm{X}}_2+\dots +{\upalpha}_{\mathrm{p}}{\mathrm{X}}_{\mathrm{p}}+\upvarepsilon \left(\mathrm{Model}\kern0.5em 1\right) $$

where α_0_ to the intercept for the population mean, α_2_X_2_ … α_p_X_p_ to the covariates we selected a priori, α_1_CpG__cbi_ correspond to *i*th methylation site in cord blood; ε is the model error term.

#### Life course analysis

Under the “developmental origins of health and disease” (DOHaD) framework [21], epigenetic endowment from the in utero environment may set the health trajectory of a developing fetus. Hence, some epigenetic marks established at birth may act independently of factors operating after birth and associate with mid-childhood serum IgE. To identify CpG sites that are differentially methylated at birth and not further modified postnatally in our cohort, we considered the following model:

$$ \log \left(\mathrm{IgE}\right)={\upbeta}_0+{\upbeta}_1\mathrm{CpG}{\_}_{\mathrm{cbi}}+{\upbeta}_2{\Delta \mathrm{CpG}}_{\mathrm{i}}+{\upbeta}_3{\mathrm{X}}_3+\dots +{\upbeta}_{\mathrm{p}}{\mathrm{X}}_{\mathrm{p}}+\upeta \left(\mathrm{Model}\kern0.5em 2\right) $$

where β_0_ is the intercept for the population mean, β_3_X_3_ … β_p_X_p_ to the baseline covariates we selected a priori, β_1_ CpG__cbi_ to the *i*th methylation site in cord blood measured at birth/baseline, and β_2_ΔCpG_i_ corresponds to the difference in DNA methylation at the *i*th CpG site between cord blood and peripheral blood (CpG__midchildhoodi_ – CpG__cbi_) measured in mid-childhood; η is the model error term.

If we frame this model in the context of a mediation analysis, the term CpG__cbi_ is the direct effect of cord blood methylation on IgE independently of postnatal change in methylation levels. We considered a methylation site to be “early programmed” if CpG__cbi_ reached epigenome-wide significance; a methylation site to capture postnatal environment and experiences if ΔCpG_i_ reached epigenome-wide significance; and a methylation site to be associated with both prenatal (CpG__cbi_) and postnatal exposure (ΔCpG_i_) if both terms remained epigenome-wide significant.

#### Sensitivity analysis

As a sensitivity analysis, we additionally adjusted for current asthma status at mid-childhood (yes/no) to examine whether the adjustment for asthma would influence our results. We additionally adjusted for asthma status in mid-childhood because (1) children with asthma may have higher total serum IgE levels compared to non-asthmatic children and (2) children maybe on asthma medication upon diagnosis, which leads to lowered serum IgE levels; for both reasons, asthma status maybe a predictor of our outcome—total serum IgE levels. On the other hand, we did not adjust for other atopic diseases (food allergy and eczema) because both of those variables showed high degree of concordance with asthma (chi-square test *p* values were both 0.0003). Including highly correlated variables may result in multi-collinearity and decrease model stability. Hence, we only adjust for asthma, a prevalence atopic phenotype in mid-childhood in our dataset (prevalence = 21.7%). We further stratified our analysis by child race/ethnicity (white, non-white) and sex to investigate whether the observed associations were consistent across population strata. We adjusted for white blood cell proportions estimated from blood reference panel [22, 23] and compared the effect estimates with the PCA-based ReFACTor method. We restricted the cord blood analysis to the 217 mother-child pairs who had DNA methylation measures at birth and mid-childhood to examine whether the discrepancy we observe between the cord blood analysis and the life course analysis were driven by a shift in participants’ characteristics. We conducted a concurrent analysis among the 210 mother-child pairs who had DNA methylation measures only during mid-childhood and compared results with the postnatal term in the life course analysis. Further, we plotted DNA methylation levels of our top associations at three different time points—birth, early childhood, and mid-childhood (*N* = 56)—to graphically illustrate the change in methylation over time. We also plotted the range of epigenome-wide significant methylation sites identified from the cord blood analysis and the life course analysis.

To investigate the genetic control of methylation for associated CpG sites, we leveraged information from a large-scale genome-wide DNA methylation quantitative trait loci (QTL) analysis of 1000 mother-child pairs to examine whether the differentially methylated sites identified were partly driven by genetic variation (http://www.mqtldb.org/) [24]. The genetic influences on DNA methylation were studied across five different time points in blood in this reference library: maternal [pregnancy, middle-age]; child [at birth, childhood, adolescence]. We focused our mQTL comparisons on the *cis* position (i.e., a genetic variant within ± 500Kb of a methylation locus) measured from cord blood and peripheral blood in childhood. We only considered SNPs with MAF ≥ 5%.

## Results

### Participants’ characteristics

Two-hundred and forty-two children had information on cord blood DNA methylation measurements and mid-childhood total serum IgE measurements (Table 1). Participants included in the current analysis did not differ substantially from the overall Project Viva study population who had serum IgE measures (Additional file 1: Table S1). Ten percent of mothers smoked during pregnancy, and 22% were former smokers (self-reported). Thirty-seven percent of mothers had a history of atopy (which includes asthma, eczema, and hay fever). One hundred and sixty-one children (67%) were white and 117 (48%) were female. Mean ± SD age at blood draw at mid-childhood was 7.8 ± 0.7 years. Total serum IgE levels showed a right-skewed distribution, with a mean ± SD of 146.4 ± 310.2 kU/L. Of the 217 children pairs who had information for both cord blood and mid-childhood DNA methylation measurements, maternal and childhood characteristics did not differ substantially from those who only had DNA methylation measured in cord blood (Table 1).

**Table 1:** Descriptive characteristics of study participants in Project Viva with information on DNA methylation and mid-childhood IgE

Participant characteristics	Cord blood DNA methylation data (*N* = 242)	Mid-childhood DNA methylation data (*N* = 411)	DNA methylation data at both time points (*N* = 217)
Maternal characteristics			
Age (years), mean ± SD	32.0 (5.6)	32.3 (5.5)	32.3 (5.4)
Smoking status, *N* (%)			
Smoked during pregnancy	24 (10%)	41 (10%)	20 (9%)
Former smoker	53 (22%)	83 (20%)	49 (23%)
Never smoker	165 (68%)	287 (70%)	148 (68%)
College graduate, *N* (%)			
Yes	158 (65%)	271 (66%)^*^	144 (66%)
No	84 (35%)	138 (34%)	73 (34%)
Atopy history			
Yes	90 (37%)	163 (40%)^*^	80 (37%)
No	152 (63%)	246 (60%)	137 (63%)
Child characteristics			
Gestational age (weeks), mean ± SD	39.7 (1.6)	39.6 (1.6)	39.7 (1.6)
Age at blood drawn (years), mean ± SD	7.8 (0.7)^†^	7.8 (0.7)^†^	7.8 (0.7)
Sex, *N* (%)			
Female	117 (48%)	201 (49%)	105 (48%)
Male	125 (52%)	210 (51%)	112 (52%)
Race/ethnicity, *N* (%)			
White	161 (67%)	256 (62%)^†^	143 (66%)
Black	42 (17%)	77 (19%)	39 (18%)
Other	39 (16%)	77 (19%)	35 (16%)
Total serum IgE level in mid-childhood, (kU/L)	146.4 (310.2)	152.1 (286.2)	142.5 (311.0)

*Number of missing = 2

^†^Number of missings = 1

### Cord blood DNA methylation and mid-childhood IgE association

**Fig. 3:**
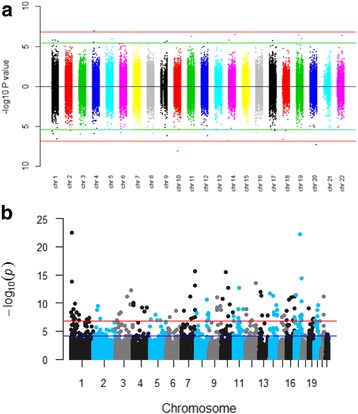
Manhattan plots. **a** Association between cord blood DNA methylation and mid-childhood total serum IgE (without—top and with—bottom adjusting for postnatal DNA methylation changes). **b** Association between changes in postnatal DNA methylation from birth and mid-childhood IgE). Red solid line—FDR significance threshold of less than 0.05; green/blue solid line—Bonferroni significance threshold of less than 0.05

Our epigenome-wide association identified 67 methylated CpG sites (representing 58 annotated genes) in cord blood associated with mid-childhood total serum IgE (FDR < 0.1) (Additional file 1: Table S2; Additional file 2: Figure S1; Fig. 3). These associations include multiple genes that have been implicated in immuno-regulation (*AOAH*, *ALOX5*, *DHX58*, and *STAM2*). Methylation loci associated with obesity (*AEBP2*), calcium signaling (*CAPNS1*), insulin signaling (*INSR*, *PTPRN2*, *RPTOR*) and vasodilation (*VASP*) also appeared to be epigenome-wide significant (Additional file 1: Table S2). With an FDR threshold of less than 0.05, 19 differentially methylated CpG sites located at 18 annotated loci in cord blood showed epigenome-wide significance (Table 2), most of which were involved in cell signaling, growth, and development. We observed minimal inflation in the epigenome-wide association analysis accounting for the first five ReFACTor components (Additional file 2: Figure S2).

**Table 2:** Association between cord blood DNA methylation and mid-childhood IgE (FDR threshold < 0.05). Results are expressed as the change in log(IgE) concentration per 1% increase in cord blood methylation value

CpG	CHR	MAPINFO	Gene	Estimate	*P* value	FDR
cg06226630	4	48,493,420	ZAR1	− 0.40	1.15E−07	0.013
cg03307893	15	26,108,683	ATP10A	− 0.76	1.20E−07	0.013
cg13322072	7	98,784,083	KPNA7	− 0.11	1.23E−07	0.013
cg16797808	14	99,948,289	SETD3;CCNK	− 0.90	3.18E−07	0.023
cg09535168	19	19,431,582	MAU2	− 1.44	3.91E−07	0.023
cg04122974	22	39,916,495	ATF4	− 3.02	4.46E−07	0.023
cg01527777	11	71,956,145	PHOX2A	− 0.24	6.06E−07	0.027
cg24575275	7	1,094,737	C7orf50	− 0.29	9.23E−07	0.032
cg14920426	5	176,924,420	PDLIM7	− 1.00	1.01E−06	0.032
cg05399209	19	46,010,836	VASP	− 1.33	1.03E−06	0.032
cg09507928	5	140,027,484	IK;NDUFA2	− 2.03	1.46E−06	0.040
cg24114890	14	23,834,349	EFS	− 0.76	1.65E−06	0.040
cg05063806	1	36,772,417	C1orf113	− 1.26	1.79E−06	0.040
cg14167858	1	120,199,593	–	0.10	1.83E−06	0.040
cg02228675	17	40,259,724	DHX58	− 0.27	1.90E−06	0.040
cg14607755	9	139,962,279	C9orf140	− 0.49	2.25E−06	0.044
cg24630419	1	217,311,608	ESRRG	− 0.41	2.49E−06	0.046
cg27212903	15	92,708,880	SLCO3A1	0.97	2.91E−06	0.048
cg14605590	9	94,900,583	LOC100128076	− 0.16	2.93E−06	0.048

Model adjusted for maternal [age at enrollment (continuous), smoking status (smoking during pregnancy/former/never), college graduate (yes/no), maternal atopy history (yes/no)], children [child’s sex (female/male), race/ethnicity (white/black/other), gestational age (continuous), age at blood drawn (continuous)], and the cell-type proxys using the first five ReFACTor components estimated from cord blood

Corresponding model: log(IgE) = α_0_ + α_1_CpG__cbi_ + α_2_X_2_ + … + α_p_X_p_ + ε

### Life course analysis

#### Prenatal influences

We identified 98 differentially methylated CpG sites (located at 82 annotated genes) associated with mid-childhood total serum IgE levels (Additional file 1: Table S3; Additional file 2: Figure S1; Fig. 3), after adjustment for postnatal DNA methylation; 13 methylation sites overlapped with the previous analysis (i.e., showed epigenome-wide significance with and without the adjustment for postnatal DNA methylation changes). The 13 methylation sites are in proximity to/within *ATP10A*, *C22orf45*, *C7orf50*, *DDO*, *INSR*, *KCNIP4*, *LOC100128076*, *MPP2*, *STAM2*, *TGIF1*, *TRIM27*, *XKR6*, and *ZAR1*, respectively (Additional file 1: Table S3). When we further restrict our analysis to an FDR threshold of less than 0.05, we identified 16 differentially methylated CpG sites (FDR < 0.05) located at 15 annotated genes in cord blood associated with mid-childhood total serum IgE levels (Table 3).

**Table 3:** Life course analysis—contribution of fetal and postnatal influences (FDR threshold < 0.05). For the prenatal analysis, results are expressed as the change in log(IgE) concentration per 1% increase in cord blood methylation value. While for the postnatal analysis, results are expressed as the change in log(IgE) concentration per 1% increase in Δ-DNAm methylation value

				Prenatal influences			Postnatal influences (Δ-DNAm)		
CpG	CHR	MAPINFO	Gene	Estimate	*P* value	FDR	Estimate	*P* value	FDR
Life course analysis—prenatal influences									
cg25854298	10	73,936,754	ASCC1	− 0.36	8.02E−09	0.003	− 0.40	1.80E−13	6.28E−09
cg16416603	20	57,593,014	TUBB1	0.58	5.17E−08	0.008	0.28	1.32E−04	8.24E−02
cg11848324	14	20,403,845	OR4K1	0.57	1.48E−07	0.016	0.44	3.35E−06	5.26E−03
cg19549714	18	3,447,713	TGIF1	− 2.00	2.40E−07	0.017	− 0.10	4.45E−01	9.71E−01
cg23933289	1	178,998,656	FAM20B	− 0.18	2.66E−07	0.017	− 0.17	8.01E-08	2.54E−04
cg24576940	7	150,648,283	KCNH2	− 1.54	3.31E−07	0.017	− 1.66	2.95E−08	1.10E−04
cg13443997	9	139,743,586	PHPT1	− 3.39	7.11E−07	0.028	− 1.83	1.50E−03	3.32E−01
cg19954205	12	122,211,282	TMEM120B	− 1.20	7.20E−07	0.028	− 0.55	1.72E−02	7.37E−01
cg01782059	1	44,715,942	ERI3	− 0.96	1.25E−06	0.033	− 0.60	4.04E−04	1.69E−01
cg02133716	8	128,981,622	PVT1	− 0.45	1.25E−06	0.033	− 0.54	2.63E−11	3.31E−07
cg06226630	4	48,493,420	ZAR1	− 0.39	1.24E−06	0.033	− 0.02	5.24E−01	9.76E−01
cg20675173	3	43,795,541	–	− 1.22	1.03E−06	0.033	− 0.72	1.11E−03	2.91E−01
cg08067346	16	25,011,481	ARHGAP17	− 0.24	1.44E−06	0.035	− 0.30	2.18E−11	2.85E−07
cg09597192	6	32,141,591	AGPAT1	− 0.31	1.78E−06	0.040	− 0.28	3.70E−06	5.67E−03
cg16096766	13	52,419,714	FLJ37307	− 0.36	2.30E−06	0.048	− 0.19	2.48E−04	1.23E−01
cg00026222	1	2,144,244	–	− 1.02	2.63E−06	0.049	− 0.29	9.59E−02	9.09E−01
cg24575275	7	1,094,737	C7orf50	− 0.31	2.49E−06	0.049	− 0.02	5.23E−01	9.76E−01
Life course analysis—postnatal influences (top 20 methylation sites)									
cg11699125	1	6,341,327	ACOT7	− 0.10	3.29E−02	5.88E-01	− 0.25	2.81E−23	7.95E−18
cg02970679	17	56,269,818	EPX	− 0.17	9.50E−04	2.86E-01	− 0.25	5.06E−23	7.95E−18
cg24491618	7	150,649,807	KCNH2	− 0.12	1.23E−03	3.05E-01	− 0.19	2.12E−16	2.22E−11
cg01614759	10	45,495,435	C10orf25	− 0.17	2.17E−03	3.46E-01	− 0.28	2.97E−16	2.33E−11
cg13054523	17	81,055,722	–	− 0.30	9.45E−05	1.53E-01	−0.43	4.23E−15	2.66E−10
cg21220721	1	6,341,230	ACOT7	0.01	7.03E−01	9.51E-01	− 0.11	1.30E−14	6.81E−10
cg10065736	12	117,440,120	FBXW8	− 0.10	2.28E−02	5.53E-01	− 0.18	2.73E−14	1.23E−09
cg06558622	7	149,543,177	ZNF862	− 0.29	3.37E−05	1.02E-01	− 0.41	8.05E−14	3.16E−09
cg25854298	10	73,936,754	ASCC1	− 0.36	8.02E−09	2.52E-03	− 0.40	1.80E−13	6.28E−09
cg05300717	11	65,546,210	DKFZp761E198	0.0002	9.93E−01	9.99E-01	− 0.33	2.11E−13	6.63E−09
cg09596645	3	181,897,670	–	− 0.15	2.74E−03	3.67E-01	− 0.25	5.56E−13	1.59E−08
cg19928703	13	30,143,971	SLC7A1	− 0.19	3.05E−03	3.75E-01	− 0.31	1.06E−12	2.78E−08
cg18368116	14	21,436,271	–	− 0.13	2.02E−02	5.40E-01	− 0.27	1.91E−12	4.62E−08
cg20885063	17	17,939,419	ATPAF2	− 0.16	1.85E−04	1.90E-01	− 0.23	4.05E−12	9.09E−08
cg08077807	14	62,001,072	PRKCH	− 0.53	7.33E−04	2.63E-01	− 0.76	4.45E−12	9.11E−08
cg07908654	13	41,631,052	–	− 0.20	3.94E−06	5.14E-02	− 0.26	4.64E−12	9.11E−08
cg08940169	16	88,540,241	ZFPM1	− 0.05	1.56E−01	7.57E-01	− 0.18	6.34E−12	1.17E−07
cg18879389	21	43,771,120	TFF2	− 0.16	6.64E−04	2.58E-01	− 0.28	6.73E−12	1.17E−07
cg20263733	3	130,616,293	ATP2C1	− 0.25	4.25E−03	3.99E-01	− 0.50	7.45E−12	1.23E−07
cg02985445	7	97,908,505	–	− 0.08	3.50E−02	5.94E-01	− 0.18	8.09E−12	1.27E−07
Life course analysis—prenatal and postnatal influences									
cg25854298	10	73,936,754	ASCC1	− 0.36	8.02E−09	0.003	− 0.40	1.80E−13	6.28E−09
cg11848324	14	20,403,845	OR4K1	0.57	1.48E−07	0.016	0.44	3.35E−06	0.005
cg23933289	1	178,998,656	FAM20B	− 0.18	2.66E−07	0.017	− 0.17	8.01E−08	2.54E−04
cg24576940	7	150,648,283	KCNH2	− 1.54	3.31E−07	0.017	− 1.66	2.95E−08	1.10 E−04
cg02133716	8	128,981,622	PVT1	− 0.45	1.25E−06	0.033	− 0.54	2.63E−11	3.31E−07
cg08067346	16	25,011,481	ARHGAP17	− 0.24	1.44E−06	0.035	− 0.30	2.18E−11	2.85E−07
cg09597192	6	32,141,591	AGPAT1	− 0.31	1.78E−06	0.040	− 0.28	3.70E−06	0.006

Model adjusted for maternal [age at enrollment (continuous), smoking status (smoking during pregnancy/former/never), college graduate (yes/no), maternal atopy history (yes/no)], children [child’s sex (female/male), race (white/black/other), gestational age (continuous), age at blood drawn (continuous)], and the cell-type proxys using the first five ReFACTor components estimated from cord blood

Corresponding model: log(IgE) = β_0_ + β_1_CpG__cbi_ + β_2_ΔCpG_i_ + β_3_X_3_ + … + β_p_X_p_ + η

*Life course analysis—prenatal influence*: association between cord blood methylation and mid-childhood IgE independent of postnatal changes in DNA methylation, which correspond to β_1_CpG__cbi_

*Life course analysis—postnatal influence*: association between changes in postnatal DNA methylation from birth and mid-childhood IgE independent of baseline/cord blood DNA methylation, which correspond to ΔCpG_i_

#### Postnatal influences (Δ-DNAm)

**Fig. 4:**
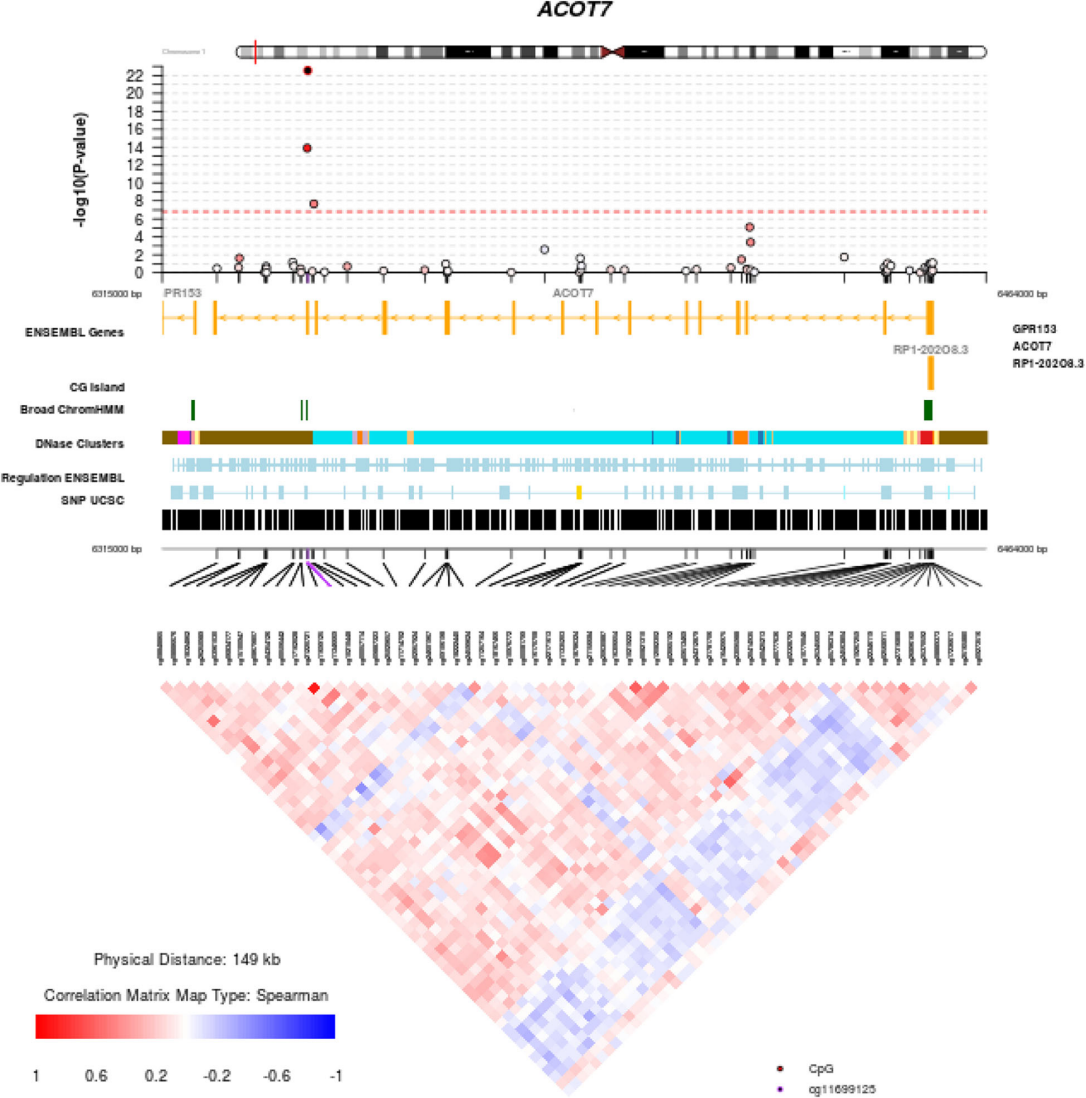
Regional manhattan plot of methylation loci within *ACOT7* gene in the lifecourse postnatal influence analysis (*y*-axis shows the raw *p* values). Dotted red line—Bonferroni significance threshold of less than 0.05

We performed an analysis of the change in methylation between cord blood and mid-childhood DNA (Δ-DNAm) and identified 395 differentially methylated sites in 272 independent loci (FDR < 0.05) in blood associated with mid-childhood serum IgE (Additional file 1: Table S4). Table 3 presents the top 20 differentially methylated sites ranked by association *p* value. *ZFPM1*, *ACOT7*, and *MDN1* have been previously associated with total serum IgE measurements in school-aged children from an independent cohort [12]. Figure 4 shows the regional plot for *ACOT7*—among the FDR-significant CpG sites identified, three of them were located within / close to CpG island of the gene body. Many of the methylation sites we identified have been associated with asthma including *ADAM19*, *EPX*, *IL4*, *IL5RA*, and *PRG2* (Table 4).

**Table 4:** Life course analysis— contribution of fetal and postnatal influences (FDR threshold < 0.05)—asthma pathway. For the prenatal analysis, results are expressed as the change in log(IgE) concentration per 1% increase in cord blood methylation value. While for the postnatal analysis, results are expressed as the change in log(IgE) concentration per 1% increase in Δ-DNAm methylation value

				Prenatal influences			Postnatal influences		
CpG	CHR	MAPINFO	Gene	Estimate	*P* value	FDR	Estimate	*P* value	FDR
Life course analysis—postnatal influences (asthma pathway)									
cg26787239	5	132,008,525	IL4	− 0.12	9.00E−02	6.91E−01	− 0.18	1.94E−05	0.020
cg01310029	3	3,152,374	IL5RA	− 0.09	7.48E−02	6.73E−01	− 0.18	3.55E−06	0.005
cg02970679	17	56,269,818	EPX	− 0.17	9.50E−04	2.86E−01	− 0.25	5.06E−23	7.95E−18
cg25173129	17	56,269,410	EPX	− 0.16	5.73E−03	4.22E−01	− 0.22	2.59E−08	9.80E−05
cg27469152	17	56,282,313	EPX	− 0.10	5.02E−02	6.32E−01	− 0.19	2.59E−07	6.53E−04
cg18421167	17	56,276,490	EPX	− 0.09	2.38E−01	8.07E−01	− 0.25	1.83E−06	0.003
cg08105265	17	56,274,480	EPX	− 0.04	6.46E−01	9.40E−01	− 0.22	1.55E−05	0.017
cg12819873	11	57,157,632	PRG2	− 0.12	8.55E−03	4.60E−01	− 0.23	1.21E−09	9.05E-06
cg15700636	11	57,156,050	PRG2	− 0.08	3.19E−02	5.85E−01	− 0.18	4.13E−08	1.46E−04
cg08295410	5	156,990,663	ADAM19	− 0.31	4.79E−03	4.09E−01	− 0.40	2.70E−05	0.026

Model adjusted for maternal [age at enrollment (continuous), smoking status (smoking during pregnancy/former/never), college graduate (yes/no), maternal atopy history (yes/no)], children [child’s sex (female/male), race (white/black/other), gestational age (continuous), age at blood drawn (continuous)], and the cell-type proxys using the first five ReFACTor components estimated from cord blood

Corresponding model: log(IgE) = β_0_ + β_1_CpG__cbi_ + β_2_ΔCpGi + β_3_X_3_ + … + β_p_X_p_ + η

*Life course analysis—prenatal influence*: association between cord blood methylation and mid-childhood IgE independent of postnatal changes in DNA methylation, which correspond to β_1_CpG__cbi_;

*Life course analysis—postnatal influence*: association between changes in postnatal DNA methylation from birth and mid-childhood IgE independent of baseline/cord blood DNA methylation, which correspond to ΔCpG_i_

#### Prenatal and postnatal influences

Twenty-two methylation sites showed epigenome-wide significance (FDR < 0.10) for both cord blood and Δ-DNAm (from birth to mid-childhood): including three mitochondrial-related genes associated with the oxidative stress pathway, namely *CS*, *SLC25A26*, and *TOMM34*. With an FDR threshold of less than 0.05, we identified seven differentially methylation sites for both cord blood and Δ-DNAm (Table 3), which include *ASCC1*, *OR4K1*, *FAM20B*, *KCNH2*, *PVT1*, *ARHGAP17*, and *AGPAT1*. In Additional file 1: Table S10, we presented summary statistics of the seven FDR-significant methylation site (FDR < 0.05). We observed higher methylation values in mid-childhood peripheral compared to cord blood among all seven methylation studied (i.e., a positive value for ΔCpG_i_). Signs of effect estimates were in the same direction for the prenatal and the postnatal terms.

### Sensitivity analysis

Our associations were robust to adjustment for current asthma status, and we observed consistent associations within non-whites and whites, and boys and girls (Additional file 1: Table S5; Additional file 1: Table S6). Further, adjusting for estimated cell proportions from blood, a reference panel did not alter estimates of our top associations identified from the primary analysis (Additional file 1: Table S7; Additional file 2: Figure S5). We observed less genomic inflation with the ReFACTor method (Additional file 2: Figures S2 and S5). Restricting the cord blood analysis to the 217 mother-child pairs who had DNA methylation measures at birth and mid-childhood also did not seem to influence our effect estimates (Additional file 1: Table S8). Among the 210 mother-child pairs who only had DNA methylation measures in mid-childhood, we observed similar effect estimates when we compared the concurrent analysis with the postnatal term of the life course analysis (Additional file 1: Table S11). Our longitudinal plots showed more changes in DNA methylation levels in the first 3 years of life (Additional file 2: Figure S3a; b; c). Even though top hits identified from the epigenome-wide association studies tend to be at the extremes of methylation (either hyper- or hypo-methylated), we still observed a number of methylation sites with greater range of methylation values (Additional file 2: Figure S4).

Among the differentially methylated CpG sites identified in the life course analysis, most of them were not associated with known genetic variants in the *cis*-position (i.e., ± 500Kb) (Additional file 1: Table S9). We identified four methylation loci with *cis*-mQTL including cg19549714 (*TGIF1*) (childhood peripheral blood), cg18879389 (*TFF2*) (cord blood and childhood peripheral blood), and cg24576940 (*KCNH2*) (cord blood and childhood peripheral blood).

## Discussion

To our knowledge, this study is the first epigenome-wide association study to conceptualize IgE-mediated allergic response under an early-childhood life-course framework using longitudinal methylation measured from birth to mid-childhood. Our data show that differential methylation patterns in cord blood are associated with total serum IgE levels in children. A number of associated cord blood methylation signals remained epigenome-wide significant after the adjustment for the change in methylation between cord blood and mid-childhood DNA (Δ-DNAm). Further, change in methylation pattern from cord blood to mid-childhood DNA (Δ-DNAm) may also be associated with total serum IgE levels—independently or in combination with prenatal methylation marks.

It is now recognized that allergic sensitization may occur as early as in utero [5, 6]. Factors such as genetic predisposition [25, 26], parental atopic status [27–29], and aspects of the intrauterine environment [30–33] including exposures to allergens in amniotic fluid [6] may impact the development of fetal immune responses and Th2 immune responses postnatally. We identified multiple differentially methylated immuno-regulatory loci in cord blood associated with total serum IgE measured in mid-childhood. For example, *AOAH* gene products—released by neutrophils and macrophages—help to neutralize and inactivate bacterial lipopolysaccharides (LPS); *ALOX5* encodes for a lipoxygenase that facilitates leukotriene synthesis—an important inflammatory mediator for allergic reaction; and *DHX58* is involved in antiviral signaling, while *STAM2* responds to cytokine stimulation in the JAK kinase signaling pathway.

In addition to the findings of these immune-regulatory genes, many other top associations have been implicated in obesity (*AEBP2*) (FDR < 0.10), calcium signaling (*CAPNS1*), insulin signaling (*INSR*, *PTPRN2*, *RPTOR*), and vasodilation (*VASP*). Abnormal insulin signaling often couples with obesity and increases the risk of asthma and other childhood allergic disorders [34–38], while calcium signaling has a long standing role in hyperpolarization of airway smooth muscle cells—activation of voltage-gated calcium channels may induce airway hyper-responsiveness—a fundamental property of asthma [39]. Although those genes may not be directly involved in immuno-regulation, alterations in these pathways during embryonic development may increase disease susceptibility and make the fetus more prone to the development of IgE-mediated allergic responses later in life.

Leveraging the longitudinal study design of our pre-birth cohort, we aimed to identify differentially methylated sites at birth that are associated with later allergic response and that may or may not be modified postnatally using changes in the epigenome from birth to childhood. We identified 98 differentially methylated CpG sites in cord blood associated with mid-childhood total serum IgE levels, which were independent of changes in DNA methylation postnatally. Thirteen of these methylation sites overlapped with the cord blood analysis where we did not adjust for methylation changes after birth. The fact that methylation levels remained epigenome-wide significant lends support to the DOHaD hypothesis, reinforcing that embryonic development is a vulnerable period with high degree of epigenetic plasticity, and exposures occurring during this period of time may embed epigenetically and could have an effect that persists for decades in life independent of postnatal/childhood influences.

Exposures during early childhood play critical roles in IgE-mediated disease onset and manifestation. We demonstrated that changes in DNA methylation levels from baseline (cord blood) until mid-childhood—potentially an “archive” [40] of the childhood environment—were associated with total serum IgE measures in school-aged children. Specifically, we identified multiple methylation loci in genes previously associated with asthma (*ADAM19*, *EPX*, *IL4*, *IL5RA*, and *PRG2*) from genome-wide and epigenome-wide association studies [10–12, 26]. Many of those methylation sites have been previously reported in independent large adult cohorts (*EPX*, *IL4*, *IL5RA*, and *PRG2*) [10, 11]. IL4 and IL5RA are well-characterized cytokines responsible for Th2 cell differentiation and effector function, ADAM19 is involved in airway and vasculature remodeling, and PRG2 and EPX are both eosinophil-related proteins that play key roles in eosinophil-related airway inflammation. PRG2 is a major protein component of the crystalline core of the eosinophil granule and is involved in epithelial cell damage, mast cell degranulation, and macrophage and neutrophil activation. EPX is a key peroxidase enzyme in the eosinophil granules, which helps to generate potent oxidizing agents directly implicated in oxidative damage processes. It is not surprising that we identified those well-established IgE-related methylation signals in the postnatal analysis—as children are exposed to a more diverse environment after birth including increased allergen exposures. Hence, we were able to identify a large number of signals related to allergic symptoms and disease manifestation. Moreover, most of these genes identified in the postnatal analysis did not overlap with the baseline signals, which suggests that the fetal environment may not necessarily be associated with disease manifestation; rather, it may help to direct the development of fetal immunity and alters disease susceptibility. Future research is warranted to explore whether the identified methylation marks are on the causal pathway linking in utero and early-life risk factors (i.e., living in rural versus urban areas, having siblings or not, attending daycare or not, antibiotic use, pets at home) and higher total serum IgE levels.

We observed that seven methylation sites showed epigenome-wide significance at both baseline and postnatally (Δ-DNAm). Among these *KCNH2* encodes for a voltage-gated K^+^ channel, *PVT1* is a candidate oncogene and *ARHGAP17* encodes for a GTPase-activating protein, which participates in the Ca^2+^ dependent regulation of exocytosis. With an FDR threshold less than 0.10, we identified 22 methylation sites (15 additional); several of these genes have been implicated in mitochondrial function (*TOMM34*, *CS*, *SLC25A26*). Mitochondria are critical cellular components that reflect and intensify oxidative stress [41]. Epigenetic alterations in nuclear-encoded mitochondrial genes at both time windows suggest that intrauterine and postnatal cellular oxidative stress may be associated with higher total serum IgE in mid-childhood. A handful of observational studies and clinical trials have shown that maternal antioxidant intake was associated with reduced allergic symptoms in children [33, 42–47], and antioxidant intake during pregnancy and early in life may potentially serves as a first-line preventive regimen to avert childhood allergy.

Since DNA methylation was measured at birth and early and mid-childhood, it would be interesting to explore how methylation patterns change in children who have persistent atopy versus those who transition to a non-atopic state. We computed a two-by-two table of atopic sensitization at early and mid-childhood. We found that 76% of children were sensitized at both time points; only 5% of children (*N* = 15) are no longer atopic by age 8. Such small sample size would make any statistical inference unreliable. It is widely accepted that there is an age-dependent allergic march during childhood [48–50], beginning with food allergy and atopic dermatitis (infancy to early-childhood), followed by asthma and rhinitis (early/mid-childhood to teen) [48–50]. Since we have methylation measurements at birth and early and mid-childhood, the ideal phenotype to study would be food allergy [51, 52]. However, food allergy was only measured at mid-childhood in Project Viva. A transition from early to mid-childhood may not be the right time window to capture subjects no longer categorized as having atopy or asthma phenotypes. Future research is needed to identify differential methylation patterns associated with transient and persistent subtypes at appropriate developmental windows.

Our study has a number of strengths including the longitudinal nature of our analyses that enabled us to conceptualize IgE-mediated disease etiology under an early-childhood life-course framework. We identified some distinct and some overlapping methylation marks at birth and postnatally that potentially reflect IgE-mediated disease sensitization, progression, and manifestation. Most of these methylation sites identified in the life course analysis were not associated with known genetic variants in the *cis*-position (i.e., ± 500Kb), and thus were likely to be related to endogenous or exogenous exposures. Our results were robust to the adjustment for batch, cell proportions, and maternal atopy history. Although we did not have external replication for our current analysis, many of our top findings have been previously reported in independent cohorts (with comparable effect estimates and direction of effect) [11, 12], suggesting that the observed associations were robust signals and are not cohort specific.

Our study has several limitations. First, we measured DNA methylation from heterogeneous blood cells at birth and in mid-childhood. Different cell types often exhibits distinct DNA methylation patterns, and if the change in relative cell abundance also correlates with total serum IgE measures in mid-childhood, then our observed associations may potentially be confounded by cellular heterogeneity. Although we adjusted for the first five PCs of ReFACTor—a stringent way to control for cell heterogeneity—we cannot rule out the possibility of residual confounding. Future analysis with relevant purified cell types might help elucidate the immune roots for the observed associations. Second, we do not have genetic information on mother-child pairs. Hence, it is difficult to disentangle the influence of genetics and the environment. Even though we studied the genetic control of selected methylation loci using mQTL information from an independent cohort with relatively large sample size (*N* = 1000) [24], obtaining genetic information from our own population will further elucidate whether the identified methylation marks reflect genetic predisposition, the impact of the intrauterine, postnatal and childhood environments, or an interaction of the two.

## Conclusion

In summary, leveraging the epigenetic plasticity from the prenatal period till childhood, our work identified differentially methylated patterns—in cord blood, as well as in changes in methylation levels from cord blood to mid-childhood DNA—associated with mid-childhood IgE. Several cord blood methylation marks associated with IgE that were independently of postnatal change in methylation levels. Further, change in methylation pattern from cord blood to mid-childhood DNA were associated with IgE—independently or in combination with cord blood methylation marks. Our study provides evidence for the epigenetic regulatory mechanism of IgE-mediated diseases and offers a scientific basis to promote early prevention of IgE-mediated diseases.

## Additional files

Additional file 1: **Table S1.** Descriptive characteristics of study participants in Project Viva. **Table S2.** Association between cord blood DNA methylation and mid-childhood IgE (FDR threshold < 0.10). **Table S3.** Life course analysis—contribution of fetal influences (FDR threshold < 0.10). **Table S4.** Life course analysis—contribution of postnatal influences (FDR threshold < 0.10). **Table S5.** Stratified analysis of methylation sites identified in the cord blood DNA methylation and mid-childhood IgE analysis. **Table S6.** Stratified analysis of methylation sites identified in the life course analysis. **Table S7.** Association between cord blood DNA methylation and mid-childhood IgE (FDR threshold < 0.05)—we estimated cell proportions using blood reference panels for cord blood. Results are expressed as the change in log(IgE) concentration per 1% increase in cord blood methylation value.** Table S8.** Association between cord blood DNA methylation and mid-childhood IgE (FDR threshold < 0.05) among the 217 children who had DNA methylation measurements both at birth and in mid-childhood. **Table S9.** Genetic influences on DNA methylation of selected top hits—results from mQTL from an independent cohort. **Table S10.** Summary statistics of selected methylation sites and their sign of associations for the prenatal and postnatal influences. **Table S11.** Association between mid-childhood peripheral blood DNA methylation and mid-childhood IgE (FDR threshold < 0.05) among the 210 children who had DNA methylation measurements both at mid-childhood only (top 20 methylation sites). (PDF 2172 kb)

Additional file 2: **Figure S1.** Scatter plot (cord blood DNA methylation and mid-childhood total serum IgE). Scatter plot (life course analysis: prenatal influence—top 6 methylation sites). Scatter plot (life course analysis: postnatal influences—top 6 methylation sites). Scatter plot (life course analysis: postnatal influences (asthma pathway)—top 6 methylation sites). Scatter plot (life course analysis—contribution of prenatal and postnatal influences—top 6 methylation sites). **Figure S2.** QQ plots. **Figure S3a.** Trend of top hits (reflect life course analysis—prenatal influence) (*N* = 56). **Figure S3b.** Trend of top hits (reflect life course analysis—prenatal and postnatal influence) (*N* = 56). **Figure S3c.** Trend of top hits (reflect life course analysis—postnatal influence) (N = 56). **Figure S4.** Distribution of the range of epigenome-wide significant methylation sites identified from the cord blood analysis and the life course analysis. **Figure S5.** QQ plot of cord blood analysis using blood reference panel to adjust for cellular heterogeneity. (PDF 330 kb)

## Abbreviations

CpG: Cytosine-guanine dinucleotide sequence
FDR: False discovery rate
IgE: Immunoglobulin E
IQR: Interquantile range
kb: Kilo-bases
MAF: Minor allele frequency
QTL: Quantitative trait locus
ReFACTor: Reference free adjustment for cell-type composition
SD: Standard deviation
SNP: Single nucleotide polymorphism
