# Supplementary material for: Epigenome-wide association study of total serum immunoglobulin E in children: a life course approach
Source: Clin Epigenetics. 2018 Apr 17;10:55. doi: 10.1186/s13148-018-0488-x (PMC5905182; doi:10.1186/s13148-018-0488-x)
Supplement: Supplementary file 1 — Table S1. Descriptive characteristics of study participants in Project Viva. Table S2. Association between cord blood DNA methylation and mid-childhood IgE (FDR threshold < 0.10). Table S3. Life course analysis—contribution of fetal influences (FDR threshold < 0.10). Table S4. Life course analysis—contribution of postnatal influences (FDR threshold < 0.10). Table S5. Stratified analysis of methylation sites identified in the cord blood DNA methylation and mid-childhood IgE analysis. Table S6. Stratified analysis of methylation sites identified in the life course analysis. Table S7. Association between cord blood DNA methylation and mid-childhood IgE (FDR threshold < 0.05)—we estimated cell proportions using blood reference panels for cord blood. Results are expressed as the change in log(IgE) concentration per 1% increase in cord blood methylation value. Table S8. Association between cord blood DNA methylation and mid-childhood IgE (FDR threshold < 0.05) among the 217 children who had DNA methylation measurements both at birth and in mid-childhood. Table S9. Genetic influences on DNA methylation of selected top hits—results from mQTL from an independent cohort. Table S10. Summary statistics of selected methylation sites and their sign of associations for the prenatal and postnatal influences. Table S11. Association between mid-childhood peripheral blood DNA methylation and mid-childhood IgE (FDR threshold < 0.05) among the 210 children who had DNA methylation measurements both at mid-childhood only (top 20 methylation sites). (PDF 2172 kb) [file 13148_2018_488_MOESM1_ESM.pdf]

Table S1. Descriptive characteristics of study participants in Project Viva.

Participant characteristics	Overall (N=616)	Cord blood (N=242)	Mid-childhood (N=411)	Both time points (N=217)
**Maternal characteristics**				
Age (years), mean ± SD	32.1 (5.5)	32.0 (5.6)	32.3 (5.5)	32.3 (5.4)
Smoking status, N (%)				
Smoked during pregnancy	61 (10%)*	24 (10%)	41 (10%)	20 (9%)
Former smoker	122 (20%)	53 (22%)	83 (20%)	49 (23%)
Never smoker	431 (70%)	165 (68%)	287 (70%)	148 (68%)
College graduate, N (%)				
Yes	407 (67%)‡	158 (65%)	271 (66%)*	144 (66%)
No	205 (33%)	84 (35%)	138 (34%)	73 (34%)
Atopy history				
Yes	241 (39%)‡	90 (37%)	163 (40%)*	80 (37%)
No	371 (61%)	152 (63%)	246 (60%)	137 (63%)
**Child characteristics**				
Gestational age (weeks), mean ± SD	39.6 (1.6)	39.7 (1.6)	39.6 (1.6)	39.7 (1.6)
Age at blood drawn (years), mean ± SD	7.8 (0.8) *	7.8 (0.7)†	7.8 (0.7)†	7.8 (0.7)
Sex, N (%)				
Female	302 (49%)	117 (48%)	201 (49%)	105 (48%)
Male	314 (51%)	125 (52%)	210 (51%)	112 (52%)
Race, N (%)				
White	383 (62%)*	161 (67%)	256 (62%)†	143 (66%)
Black	121 (20%)	42 (17%)	77 (19%)	39 (18%)
Other	110 (18%)	39 (16%)	77 (19%)	35 (16%)
Total serum IgE level, (kU/L)	149.8 (296.4)	146.4 (310.2)	152.1 (286.2)	142.5 (311.0)

* number of missings=2; † number of missings =1; ‡ number of missings=4

Table S2. Association between cord blood DNA methylation and mid-childhood IgE (FDR threshold <0.10).
Results are expressed as the change in log(IgE) level per 1% increase in cord blood methylation value.

CpG	CHR	MAPINFO	Gene	Estimate	P value	FDR
cg0622663	4	48493420	ZAR1	-0.4	1.15E-07	0.013
cg0330789	15	26108683	ATP10A	-0.76	1.20E-07	0.013
cg1332207	7	98784083	KPNA7	-0.11	1.23E-07	0.013
cg1679780	14	99948289	SETD3;CCN	-0.9	3.18E-07	0.023
cg0953516	19	19431582	MAU	-1.44	3.91E-07	0.023
cg0412297	22	39916495	ATF4	-3.02	4.46E-07	0.023
cg0152777	11	71956145	PHOX2A	-0.24	6.06E-07	0.027
cg2457527	7	1094737	C7orf50	-0.29	9.23E-07	0.032
cg1492042	5	1.77E+08	PDLIM7	-1	1.01E-06	0.032
cg0539920	19	46010836	VASP	-1.33	1.03E-06	0.032
cg0950792	5	1.4E+08	IK;NDUFA2	-2.03	1.46E-06	0.04
cg2411489	14	23834349	EFS	-0.76	1.65E-06	0.04
cg0506380	1	36772417	C1orf113	-1.26	1.79E-06	0.04
cg1416785	1	1.2E+08	NA	0.1	1.83E-06	0.04
cg0222867	17	40259724	DHX58	-0.27	1.90E-06	0.04
cg1460775	9	1.4E+08	C9orf140	-0.49	2.25E-06	0.044
cg2463041	1	2.17E+08	ESRRG	-0.41	2.49E-06	0.046
cg2721290	15	92708880	SLCO3A1	0.97	2.91E-06	0.048
cg1460559	9	94900583	LOC10012{	-0.16	2.93E-06	0.048
cg0068896	4	21950567	KCNIP4	-0.7	3.32E-06	0.051
cg2497558	12	1.21E+08	MLEC	0.63	3.44E-06	0.051
cg2546124	8	1019317	NA	0.62	4.13E-06	0.059
cg0673341	6	85473344	TBX18	-0.49	4.36E-06	0.06
cg1833593	6	2765945	WRNIP1	-2.49	4.72E-06	0.062
cg1105105	8	11058145	XKR6	-0.59	5.26E-06	0.066
cg0125265	19	7198410	INSR	-0.61	5.70E-06	0.069
cg2744811	7	1.57E+08	PTPRN2	-0.91	6.23E-06	0.071
cg2366431	19	36630914	CAPNS1	-2.92	6.33E-06	0.071
cg0226569	7	36711771	AOAH	-0.95	6.63E-06	0.071
cg1161324	19	30302405	CCNE1	-0.62	6.81E-06	0.071
cg0776796	17	41978060	MPP2	-1.16	7.74E-06	0.076
cg2207694	1	70036106	NA	-0.04	7.97E-06	0.076
cg2248826	7	1498641	MICALL2	-0.39	7.97E-06	0.076
cg1164553	6	1.11E+08	DDO	-0.02	9.30E-06	0.077
cg2556490	17	17140463	FLCN	-0.65	9.32E-06	0.077
cg0108857	6	1.43E+08	GPR126	-0.51	9.39E-06	0.077
cg0441368	6	71998398	OGFRL1	-0.59	9.67E-06	0.077
cg0327034	6	28891204	TRIM27	-0.89	1.00E-05	0.077
cg1956075	1	8086721	ERRFI1	-0.34	1.01E-05	0.077
cg2043327	1	1.56E+08	NA	-1.88	1.01E-05	0.077
cg2324059	11	47296545	MADD	2.15	1.01E-05	0.077
cg0559675	12	47610220	FAM113B	0.12	1.07E-05	0.079
cg1948047	2	559042	NA	0.41	1.08E-05	0.079
cg2248884	5	3607134	NA	-0.15	1.15E-05	0.082
cg2658586	7	1.49E+08	ZNF767	-0.76	1.17E-05	0.082
cg2170051	20	3713032	HSPA12B	-0.09	1.24E-05	0.082
cg0966717	5	1.72E+08	NEURL1B	-0.49	1.25E-05	0.082
cg2062533	2	1.53E+08	STAM2	-0.08	1.29E-05	0.082

cg2128976	17	78868835	RPTOR	0.22	1.31E-05	0.082
cg0266421	12	19592651	AEBP2	-0.71	1.32E-05	0.082
cg0262836	17	79315269	NA	-0.23	1.35E-05	0.082
cg1555284	22	24890809	C22orf45;L	-0.34	1.36E-05	0.082
cg2704003	16	325184	RGS11	-0.5	1.40E-05	0.083
cg2238547	4	1.86E+08	CCDC110	-0.64	1.42E-05	0.083
cg1904770	2	2.33E+08	ECEL1	-0.42	1.45E-05	0.083
cg0095750	17	81054294	NA	0.28	1.50E-05	0.083
cg2208505	6	27661905	NA	-0.44	1.51E-05	0.083
cg0023533	4	42117223	BEND4	-0.27	1.54E-05	0.083
cg2624347	13	51484009	RNASEH2B	-1.95	1.63E-05	0.087
cg2321090	10	45938664	ALOX5	0.3	1.77E-05	0.093
cg1577004	19	35764710	USF2	0.13	1.80E-05	0.093
cg0113867	6	32130686	PPT2	-0.99	1.86E-05	0.094
cg1034003	2	2.2E+08	ZFAND2B	-0.56	1.91E-05	0.094
cg2171133	16	1425548	UNKL	0.32	1.91E-05	0.094
cg0113870	2	2.19E+08	TNS1	0.02	1.94E-05	0.094
cg1954971	18	3447713	TGIF1	-1.71	2.01E-05	0.096
cg0897895	1	1.2E+08	ZNF697	-0.62	2.12E-05	0.099

Model adjusted for maternal [age at enrollment (continuous), smoking status (smoking during pregnancy / former / never), college graduate (yes / no), maternal atopy history (yes / no)], children [child's sex (female / male), race (white / black / other), gestational age (continuous), age at blood drawn (continuous)], and the first five ReFACTor components.

Table S3. Life course analysis—contribution of fetal influences (FDR threshold <0.10).
Results are expressed as the change in log(IgE) concentration per 1% increase in cord blood methylation value.

CpG	CHR	MAPINFO	Gene	Estimate	P value	FDR
cg2585429	10	73936754	ASCC1	-0.36	8.02E-09	0.003
cg1641660	20	57593014	TUBB1	0.58	5.17E-08	0.008
cg1184832	14	20403845	OR4K1	0.57	1.48E-07	0.016
cg1954971	18	3447713	TGIF1	-2	2.40E-07	0.017
cg2393328	1	1.79E+08	FAM20B	-0.18	2.66E-07	0.017
cg2457694	7	1.51E+08	KCNH2	-1.54	3.31E-07	0.017
cg1344399	9	1.4E+08	PHPT1	-3.39	7.11E-07	0.028
cg1995420	12	1.22E+08	TMEM120E	-1.2	7.20E-07	0.028
cg2067517	3	43795541	NA	-1.22	1.03E-06	0.033
cg0622663	4	48493420	ZAR1	-0.39	1.24E-06	0.033
cg0178205	1	44715942	ERI3	-0.96	1.25E-06	0.033
cg0213371	8	1.29E+08	PVT1	-0.45	1.25E-06	0.033
cg0806734	16	25011481	ARHGAP17	-0.24	1.44E-06	0.035
cg0959719	6	32141591	AGPAT1	-0.31	1.78E-06	0.04
cg1609676	13	52419714	FLJ37307	-0.36	2.30E-06	0.048
cg2457527	7	1094737	C7orf50	-0.31	2.49E-06	0.049
cg0002622	1	2144244	NA	-1.02	2.63E-06	0.049
cg0017889	2	1.75E+08	NA	1.43	2.96E-06	0.05
cg1575650	17	65471461	PITPNC1	-0.1	3.09E-06	0.05
cg2092371	18	32557027	MAPRE2	-1.71	3.21E-06	0.05
cg0360359	8	29442834	NA	-0.45	3.37E-06	0.05
cg2402882	12	56694932	CS	-0.82	3.87E-06	0.051
cg0790865	13	41631052	NA	-0.2	3.94E-06	0.051
cg1859662	17	65471321	PITPNC1	-0.08	4.04E-06	0.051
cg1681977	10	1.03E+08	NA	-1.19	4.23E-06	0.051
cg0960704	16	14402157	MIR365-1	-0.21	4.39E-06	0.051
cg0338306	17	21414968	NA	-1.9	4.42E-06	0.051
cg1407796	14	53418979	FERMT2	-0.23	5.43E-06	0.059
cg0327034	6	28891204	TRIM27	-1.12	5.51E-06	0.059
cg1975904	5	79135093	NA	0.88	5.96E-06	0.059
cg1909063	4	1.11E+08	ELOVL6	-1.57	5.97E-06	0.059
cg1413097	22	19711023	GP1BB;SEP	-1.83	6.25E-06	0.059
cg2416645	19	45590882	GEMIN7	0.18	6.41E-06	0.059
cg0196550	1	2.07E+08	PIGR	0.28	6.59E-06	0.059
cg2405971	21	45138843	PDXK	-1.74	6.86E-06	0.059
cg2527042	7	24965657	OSBPL3	-0.56	6.89E-06	0.059
cg2601954	8	1.2E+08	TNFRSF11E	0.55	6.93E-06	0.059
cg1460559	9	94900583	LOC10012[illegible]	-0.16	7.69E-06	0.064
cg2638779	7	1.5E+08	GIMAP8	-1.38	8.24E-06	0.064
cg1164553	6	1.11E+08	DDO	-0.02	8.44E-06	0.064
cg1105105	8	11058145	XKR6	-0.59	8.59E-06	0.064
cg0523782	20	39945987	NA	-0.9	8.80E-06	0.064
cg0541214	10	3827605	KLF6	-5.14	8.82E-06	0.064

cg1379258	20	43590115	TOMM34	-0.42	9.04E-06	0.064
cg0330789	15	26108683	ATP10A	-0.97	9.12E-06	0.064
cg1461125	17	17946468	C17orf39	-0.38	9.35E-06	0.064
cg1197974	20	814510	FAM110A	-0.84	9.53E-06	0.064
cg0531496	21	45393393	AGPAT3	0.71	9.93E-06	0.065
cg2188661	19	8274513	LASS4	-0.98	1.01E-05	0.065
cg1555284	22	24890809	C22orf45;U	-0.33	1.06E-05	0.067
cg2266611	6	30182186	TRIM26	-0.84	1.09E-05	0.067
cg1904114	11	1.18E+08	TMEM25	-0.56	1.14E-05	0.067
cg2382074	8	1.45E+08	ZC3H3	0.6	1.14E-05	0.067
cg0889886	17	13941263	NA	0.14	1.16E-05	0.067
cg1732309	12	1.11E+08	IFT81	0.18	1.20E-05	0.069
cg2577009	5	39425547	DAB2	-0.33	1.31E-05	0.073
cg1736448	16	30381677	TBC1D10B	-1.7	1.32E-05	0.073
cg0962636	16	4524711	NMRAL1;H	-1.01	1.37E-05	0.073
cg1132802	20	34207568	SPAG4	-1.8	1.42E-05	0.073
cg1741110	17	65241599	HELZ	-2.06	1.45E-05	0.073
cg2217670	3	1.94E+08	NA	-0.1	1.47E-05	0.073
cg0096887	13	1.14E+08	F10	0.14	1.49E-05	0.073
cg0629248	3	1.83E+08	DCUN1D1	1.13	1.53E-05	0.073
cg0754774	6	1.28E+08	NA	0.83	1.54E-05	0.073
cg2258854	2	2.28E+08	MFF	-2.6	1.54E-05	0.073
cg2062533	2	1.53E+08	STAM2	-0.09	1.55E-05	0.073
cg0776796	17	41978060	MPP2	-1.47	1.57E-05	0.073
cg1297223	9	91605893	C9orf47	-0.23	1.60E-05	0.073
cg1410160	12	6797953	ZNF384	-0.94	1.60E-05	0.073
cg1972598	11	67007604	KDM2A	-0.93	1.69E-05	0.075
cg2485297	3	59352714	NA	0.58	1.72E-05	0.075
cg2148422	3	32859417	TRIM71	-1.98	1.73E-05	0.075
cg0741059	3	66404129	SLC25A26	-0.28	1.75E-05	0.075
cg0557645	11	1.26E+08	HYLS1	-0.41	1.87E-05	0.079
cg2250951	5	1.23E+08	CSNK1G3	-3.01	1.89E-05	0.079
cg1297393	19	45755125	MARK4	-0.9	1.96E-05	0.081
cg0125265	19	7198410	INSR	-0.92	2.02E-05	0.082
cg1101492	3	36974725	TRANK1	-0.47	2.05E-05	0.083
cg2180900	19	6739524	TRIP10	-1.18	2.16E-05	0.084
cg2280658	15	75871813	PTPN9	-2.05	2.19E-05	0.084
cg0010292	6	18156401	TPMT;KDM	-2.42	2.20E-05	0.084
cg0755102	1	2.06E+08	SLC41A1	2.17	2.20E-05	0.084
cg0068896	4	21950567	KCNIP4	-0.66	2.24E-05	0.085
cg2324479	5	1.41E+08	PCDHGA4	-0.85	2.28E-05	0.085
cg0906819	2	65357064	RAB1A	-0.88	2.33E-05	0.086
cg2203698	3	1.41E+08	SPSB4	-0.68	2.38E-05	0.087
cg2354076	19	1173635	SBNO2	-1.17	2.40E-05	0.087
cg2294697	1	1.53E+08	SPRR2F	0.5	2.45E-05	0.087
cg1375494	7	1.56E+08	NA	0.28	2.47E-05	0.087
cg1086180	17	40575499	PTRF	-0.99	2.68E-05	0.094

cg0155393	1	46641061	TSPAN1	0.19	2.79E-05	0.095
cg0779737	12	44316362	TMEM117	0.87	2.79E-05	0.095
cg2647384	5	37834909	GDNF	-0.55	2.82E-05	0.095
cg0052864	2	97536287	SEMA4C	-3.56	2.91E-05	0.096
cg0620780	1	44398998	ARTN	-0.27	2.94E-05	0.096
cg1517947	15	70736858	NA	-0.72	2.94E-05	0.096
cg0691328	2	18742029	RDH14	-0.74	2.99E-05	0.096
cg1269162	1	53152081	C1orf163	-2.25	2.99E-05	0.096

Model adjusted for maternal [age at enrollment (continuous), smoking status (smoking during pregnancy / former / never), college graduate (yes / no), maternal atopy history (yes / no)], children [child’s sex (female / male), race (white / black / other), gestational age (continuous), age at blood drawn (continuous)], and the first five ReFACTor components.

Table S4. Life course analysis—contribution of postnatal influences (FDR threshold <0.10).
Results are expressed as the change in log(IgE) concentration per 1% increase in delta-DNAm methylation value.

CpG	CHR	MAPINFO	Gene	Estimate	P value	FDR
cg1169912	1	6341327	ACOT7	-0.25	2.81E-23	7.95E-18
cg0297067	17	56269818	EPX	-0.25	5.06E-23	7.95E-18
cg2449161	7	1.51E+08	KCNH2	-0.19	2.12E-16	2.22E-11
cg0161475	10	45495435	C10orf25;Z	-0.28	2.97E-16	2.33E-11
cg1305452	17	81055722	NA	-0.43	4.23E-15	2.66E-10
cg2122072	1	6341230	ACOT7	-0.11	1.30E-14	6.81E-10
cg1006573	12	1.17E+08	FBXW8	-0.18	2.73E-14	1.23E-09
cg0655862	7	1.5E+08	ZNF862	-0.41	8.05E-14	3.16E-09
cg2585429	10	73936754	ASCC1	-0.4	1.80E-13	6.28E-09
cg0530071	11	65546210	DKFZp761E	-0.33	2.11E-13	6.63E-09
cg0959664	3	1.82E+08	NA	-0.25	5.56E-13	1.59E-08
cg1992870	13	30143971	SLC7A1	-0.31	1.06E-12	2.78E-08
cg1836811	14	21436271	NA	-0.27	1.91E-12	4.62E-08
cg2088506	17	17939419	ATPAF2	-0.23	4.05E-12	9.09E-08
cg0807780	14	62001072	PRKCH	-0.76	4.45E-12	9.11E-08
cg0790865	13	41631052	NA	-0.26	4.64E-12	9.11E-08
cg0894016	16	88540241	ZFPM1	-0.18	6.34E-12	1.17E-07
cg1887938	21	43771120	TFF2;TFF2	-0.28	6.73E-12	1.17E-07
cg2026373	3	1.31E+08	ATP2C1	-0.5	7.45E-12	1.23E-07
cg0298544	7	97908505	NA	-0.18	8.09E-12	1.27E-07
cg1358762	9	95497984	BICD2;BICD	-0.34	8.84E-12	1.32E-07
cg0199878	16	55542709	LPCAT2	-0.18	1.07E-11	1.53E-07
cg0521942	10	11291279	CUGBP2	-0.21	1.70E-11	2.32E-07
cg0806734	16	25011481	ARHGAP17	-0.3	2.18E-11	2.85E-07
cg0213371	8	1.29E+08	PVT1	-0.54	2.63E-11	3.31E-07
cg2674930	17	81043782	METRNL	-0.67	5.58E-11	6.74E-07
cg0573664	4	3123132	HTT	-0.42	1.04E-10	1.21E-06
cg0682419	1	47157809	KIAA0494	-0.3	1.42E-10	1.59E-06
cg0879939	10	97049193	PDLIM1	-0.2	1.57E-10	1.70E-06
cg0869868	3	1.71E+08	TNIK	-0.24	2.03E-10	2.13E-06
cg1379258	20	43590115	TOMM34	-0.59	2.21E-10	2.24E-06
cg0190157	14	95615731	DICER1	-0.18	2.30E-10	2.26E-06
cg0407708	4	9937674	SLC2A9	-0.33	2.69E-10	2.56E-06
cg0652881	2	47242277	TTC7A	-0.16	3.03E-10	2.80E-06
cg1461296	9	1.31E+08	SLC25A25	-0.26	3.15E-10	2.83E-06
cg1261452	4	1.54E+08	MND1	-0.13	6.24E-10	5.45E-06
cg2639681	4	1.03E+08	BANK1	-0.3	7.11E-10	5.90E-06
cg0349209	9	91994786	SEMA4D	-0.13	7.13E-10	5.90E-06
cg0498368	16	88558223	ZFPM1	-0.1	8.25E-10	6.65E-06
cg1833728	19	930871	ARID3A	-0.32	8.65E-10	6.79E-06
cg1617769	1	27240669	NR0B2	-0.18	1.11E-09	8.51E-06
cg1281987	11	57157632	PRG2	-0.23	1.21E-09	9.05E-06
cg1507589	12	7862691	DPPA3	-0.46	1.24E-09	9.06E-06

cg2436896	11	1.12E+08	SIK2	-0.52	1.39E-09	9.93E-06
cg1866645	7	1.51E+08	KCNH2	-0.2	1.57E-09	1.06E-05
cg1357685	9	97403129	FBP1	-0.15	1.58E-09	1.06E-05
cg0369309	9	1.36E+08	CEL	-0.16	1.66E-09	1.06E-05
cg0797094	7	1.5E+08	ZNF862	-0.18	1.67E-09	1.06E-05
cg2031385	2	38825345	HNRPLL	-0.21	1.68E-09	1.06E-05
cg0041407	20	35504511	C20orf118	-0.16	1.69E-09	1.06E-05
cg1640945	14	1.01E+08	EVL	-0.19	1.77E-09	1.09E-05
cg1145601	6	1.11E+08	NA	-0.17	2.05E-09	1.24E-05
cg0449799	16	616212	NHLRC4	-0.17	2.29E-09	1.36E-05
cg2527042	7	24965657	OSBPL3	-0.59	2.47E-09	1.44E-05
cg1723345	4	1.3E+08	PHF17	-0.52	2.98E-09	1.70E-05
cg0217078	14	69650830	NA	-0.16	4.14E-09	2.32E-05
cg2686549	10	1.29E+08	DOCK1	-0.27	4.39E-09	2.38E-05
cg1659981	1	2036283	PRKCZ	-0.16	4.39E-09	2.38E-05
cg1070417	1	62209607	INADL	-0.18	4.75E-09	2.53E-05
cg0741059	3	66404129	SLC25A26	-0.31	5.17E-09	2.71E-05
cg2654705	8	1.42E+08	NA	-0.14	5.98E-09	3.08E-05
cg2191972	8	11719367	CTSB	-0.15	7.12E-09	3.61E-05
cg1514656	3	42948626	ZNF662	-0.12	8.33E-09	4.15E-05
cg0224553	17	81043668	METRNL	-0.2	1.00E-08	4.88E-05
cg0073668	8	1.35E+08	ST3GAL1	-0.22	1.01E-08	4.88E-05
cg1943493	12	7104184	LPCAT3	-0.15	1.06E-08	5.05E-05
cg2679124	1	87596895	LOC33952	-0.16	1.13E-08	5.30E-05
cg1126739	21	45815264	TRPM2	-0.23	1.20E-08	5.54E-05
cg0094430	5	60142446	NA	-0.17	1.23E-08	5.60E-05
cg0455729	14	70119254	KIAA0247	-0.16	1.34E-08	6.01E-05
cg1855084	14	1.01E+08	EVL	-0.16	1.36E-08	6.02E-05
cg1626372	1	29523841	MECR	-0.17	1.50E-08	6.55E-05
cg0145355	8	1.44E+08	NA	-0.51	1.61E-08	6.93E-05
cg0672528	17	80533762	FOXK2	-0.26	1.65E-08	7.01E-05
cg1166814	15	72636405	HEXA	-0.29	1.69E-08	7.06E-05
cg2641543	14	1.01E+08	EVL	-0.28	1.73E-08	7.06E-05
cg0690686	13	52734154	NEK3	-0.17	1.73E-08	7.06E-05
cg1217216	13	1.14E+08	PROZ	-0.15	1.82E-08	7.33E-05
cg0626393	11	916170	CHID1	-0.35	2.08E-08	8.27E-05
cg1021044	1	6342435	ACOT7	-0.23	2.17E-08	8.52E-05
cg0036603	10	76781121	MYST4	-0.51	2.38E-08	9.23E-05
cg1198872	20	33416959	NA	-0.12	2.41E-08	9.23E-05
cg2517312	17	56269410	EPX	-0.22	2.59E-08	9.80E-05
cg2457694	7	1.51E+08	KCNH2	-1.66	2.95E-08	0.00011
cg2623549	17	1047567	ABR;ABR	-0.17	3.10E-08	0.000115
cg1240231	15	52135487	TMOD3	-0.24	3.31E-08	0.000121
cg1892790	6	36238043	PNPLA1	-0.17	3.43E-08	0.000124
cg0006815	1	2.01E+08	CACNA1S	-0.25	3.78E-08	0.000135
cg1570063	11	57156050	PRG2	-0.18	4.13E-08	0.000146
cg1597559	7	1.38E+08	CREB3L2	-0.43	4.32E-08	0.000151

cg0294185	7	1.05E+08	SRPK2	-0.32	5.09E-08	0.000175
cg1798818	2	74612222	LOC10018	-0.16	5.11E-08	0.000175
cg0310105	17	71258936	CPSF4L	-0.24	5.68E-08	0.000192
cg0921211	1	42630526	GUCA2A	-0.15	6.04E-08	0.000202
cg0242783	19	51961937	SIGLEC8	-0.18	6.18E-08	0.000204
cg2253223	19	40229215	CLC	-0.13	7.52E-08	0.000245
cg1636214	5	10708717	DAP	-0.16	7.57E-08	0.000245
cg0937753	8	1.41E+08	TRAPPC9	-0.27	7.82E-08	0.000251
cg2393328	1	1.79E+08	FAM20B	-0.17	8.01E-08	0.000254
cg0852070	8	1.42E+08	DENND3	-0.27	9.44E-08	0.000297
cg1913166	3	17236940	TBC1D5	-0.12	9.63E-08	0.0003
cg0791445	1	27241853	NR0B2	-0.17	9.89E-08	0.000305
cg0956531	5	1.13E+08	MCC	-0.22	1.05E-07	0.000314
cg2568949	17	81042554	METRNL	-0.16	1.05E-07	0.000314
cg0686620	16	67560245	NA	-0.2	1.05E-07	0.000314
cg0453858	12	71835737	LGR5	-0.33	1.06E-07	0.000314
cg1497824	5	79501131	SERINC5	-0.16	1.09E-07	0.00032
cg0179881	17	3906674	NA	-0.43	1.15E-07	0.000335
cg2645701	19	55740188	TMEM86B	-0.17	1.17E-07	0.000337
cg0161307	20	4764277	RASSF2	-0.43	1.18E-07	0.000337
cg0247569	16	616220	NHLRC4	-0.15	1.23E-07	0.000348
cg0778199	1	87655840	NA	-0.21	1.25E-07	0.000351
cg1319755	20	60709957	LSM14B	-0.51	1.46E-07	0.000402
cg0110430	11	4066205	STIM1	-0.12	1.46E-07	0.000402
cg0615490	11	64642558	EHD1	-0.12	1.47E-07	0.000402
cg1960964	8	1.25E+08	NA	-0.19	1.72E-07	0.000466
cg2402882	12	56694932	CS	-0.85	1.95E-07	0.000524
cg1944829	20	35504064	C20orf118	-0.15	2.01E-07	0.000535
cg1534464	5	1.77E+08	LMAN2	-0.34	2.16E-07	0.00057
cg1628887	1	2.06E+08	RAB7L1	-0.86	2.22E-07	0.000581
cg0864047	16	85551478	NA	-0.14	2.24E-07	0.000582
cg0945842	2	2.32E+08	CAB39	-0.19	2.52E-07	0.000646
cg0133099	17	7648108	DNAH2	-0.15	2.53E-07	0.000646
cg2746915	17	56282313	EPX	-0.19	2.59E-07	0.000653
cg1375318	9	32989852	APTX	-0.25	2.60E-07	0.000653
cg0924188	20	35504371	C20orf118	-0.17	2.62E-07	0.000653
cg1758277	1	1.55E+08	EFNA3	-0.29	2.77E-07	0.000685
cg1548271	11	70053254	FADD	-0.17	2.81E-07	0.00069
cg1797125	1	1.78E+08	SEC16B	-0.2	2.85E-07	0.000694
cg0717786	15	52030746	LYSMD2	-0.16	2.88E-07	0.000696
cg1752112	19	51961666	SIGLEC8	-0.2	3.02E-07	0.000724
cg0349312	5	1.77E+08	B4GALT7	-0.24	3.16E-07	0.000752
cg2487634	4	56798053	NA	-0.38	3.21E-07	0.000758
cg0665506	5	1.79E+08	MGAT4B	-0.18	3.47E-07	0.000814
cg0781901	11	46619375	NA	-0.14	3.97E-07	0.000924
cg2578021	3	35785041	MIR128-2	-0.2	4.24E-07	0.000979
cg1980516	1	1.6E+08	CCDC19	-0.19	4.27E-07	0.000979

cg1177032	13	80066032	NDFIP2	-0.13	4.66E-07	0.001061
cg1362844	9	1.35E+08	MED27	-0.15	4.72E-07	0.001067
cg1461387	20	35504198	C20orf118	-0.15	5.02E-07	0.001125
cg0240242	19	41255444	C19orf54;S	-0.16	5.05E-07	0.001125
cg2625207	1	61607055	NFIA	-0.14	5.24E-07	0.001159
cg2123931	7	23640510	CCDC126	-0.15	5.99E-07	0.001316
cg0867901	19	12997997	KLF1	-0.18	6.16E-07	0.001344
cg0639141	3	71295684	FOXP1	-0.19	6.38E-07	0.001383
cg1897234	1	28195327	NA	-0.23	6.59E-07	0.001415
cg0429013	7	65439512	GUSB	-0.31	6.62E-07	0.001415
cg2291953	3	48334413	NA	-0.19	6.85E-07	0.001454
cg0372335	17	46047760	CDK5RAP3	-0.12	6.93E-07	0.001461
cg1364529	15	64275810	DAPK2	-0.16	7.01E-07	0.001468
cg0877694	16	11729131	NA	-0.14	7.16E-07	0.00149
cg2050332	9	1.02E+08	COL15A1	-0.73	7.21E-07	0.00149
cg0697290	16	30488321	ITGAL	-0.11	7.92E-07	0.001618
cg1348065	1	4795012	AJAP1	-0.93	7.93E-07	0.001618
cg2222157	3	1.96E+08	PCYT1A	-0.18	7.98E-07	0.001618
cg1620576	20	4764312	RASSF2	-0.75	8.47E-07	0.001706
cg2728812	12	1.11E+08	HVCN1	-0.23	8.80E-07	0.001761
cg1590973	3	69811645	MITF	-0.48	8.92E-07	0.001774
cg0933250	1	1.6E+08	COPA	-0.63	9.29E-07	0.001828
cg2281634	8	26243601	BNIP3L	-0.29	9.31E-07	0.001828
cg1924703	21	43786727	TFF1	-0.34	9.37E-07	0.001829
cg2405880	1	66777579	PDE4B	-0.21	1.00E-06	0.00194
cg2351668	10	1.04E+08	NOLC1	-0.14	1.03E-06	0.001985
cg0992918	12	92501570	NA	-0.19	1.04E-06	0.001993
cg1665819	10	71075744	HK1	-0.52	1.07E-06	0.002025
cg1420171	7	47679990	NA	0.51	1.07E-06	0.002025
cg0664878	3	1.05E+08	ALCAM	-0.4	1.11E-06	0.002088
cg1622580	8	1.27E+08	NA	-0.12	1.12E-06	0.002095
cg2646647	16	15993454	NA	-0.13	1.20E-06	0.002231
cg2037900	12	53271818	NA	-0.14	1.21E-06	0.002236
cg1975984	17	56283364	MKS1	-0.15	1.25E-06	0.002297
cg1043354	17	41472748	NA	-0.17	1.35E-06	0.002466
cg2677497	6	1.59E+08	TMEM181	-0.24	1.37E-06	0.002488
cg0763622	11	63466987	RTN3	-0.22	1.40E-06	0.002514
cg2410850	1	1.79E+08	FAM20B	-0.12	1.40E-06	0.002514
cg0894564	5	205863	CCDC127	-0.35	1.44E-06	0.002571
cg1979883	7	1.4E+08	PARP12	-0.11	1.54E-06	0.002734
cg0427941	1	2.31E+08	NA	0.09	1.61E-06	0.002842
cg2685198	5	1.26E+08	NA	-0.12	1.74E-06	0.003054
cg1325865	1	45244184	RPS8	-0.27	1.77E-06	0.00309
cg1101492	3	36974725	TRANK1	-0.47	1.83E-06	0.003159
cg1842116	17	56276490	EPX	-0.25	1.83E-06	0.003159
cg2635271	7	36024391	NA	-0.19	1.85E-06	0.003176
cg2314485	3	1.93E+08	OPA1	-0.3	1.87E-06	0.003193

cg2487257	19	41129881	LTBP4	-0.29	1.99E-06	0.00338
cg2636938	7	1E+08	TFR2	-0.11	2.04E-06	0.003446
cg0164964	20	35177530	MYL9	-0.18	2.06E-06	0.003461
cg1773884	2	31094363	NA	-0.15	2.08E-06	0.003475
cg0469250	11	47401027	SPI1	-0.16	2.09E-06	0.003475
cg0088137	15	1.02E+08	NA	-0.46	2.45E-06	0.00403
cg1604666	20	35422698	C20orf117	-0.14	2.45E-06	0.00403
cg2186123	8	1.42E+08	SLC45A4	-0.23	2.49E-06	0.004075
cg2387127	7	1577697	MAFK	-0.19	2.65E-06	0.004314
cg1425248	1	61331027	NA	-0.49	2.75E-06	0.004454
cg0002110	14	92980026	RIN3	-1.19	2.79E-06	0.004496
cg0097878	17	71185631	NA	-0.2	2.84E-06	0.004553
cg1221368	6	24877462	FAM65B	-0.29	3.28E-06	0.005231
cg2323426	14	55582407	NA	-0.18	3.30E-06	0.005237
cg2438065	13	31308509	ALOX5AP	-0.23	3.33E-06	0.005258
cg1184832	14	20403845	OR4K1	0.44	3.35E-06	0.005263
cg0372733	10	52379467	SGMS1	-0.18	3.50E-06	0.005471
cg0659741	11	69251883	NA	-0.12	3.53E-06	0.005491
cg0131002	3	3152374	IL5RA	-0.18	3.55E-06	0.005495
cg2089191	7	1.12E+08	IFRD1	-0.22	3.60E-06	0.005545
cg0959719	6	32141591	AGPAT1	-0.28	3.70E-06	0.005671
cg1754076	17	73631586	RECQL5;LO	-0.11	3.94E-06	0.00601
cg0186483	14	55583639	NA	-0.14	3.99E-06	0.006056
cg1168348	1	44678623	DMAP1	-0.12	4.09E-06	0.006178
cg1281869	6	24877582	FAM65B	-0.18	4.13E-06	0.006209
cg1999828	12	1.22E+08	RNF34	-0.12	4.25E-06	0.006344
cg2081346	7	2646259	IQCE	-0.14	4.26E-06	0.006344
cg1113770	14	21508979	RNASE7	-0.19	4.62E-06	0.006827
cg1331233	2	74612759	LOC10018[illegible]	-0.14	4.64E-06	0.006827
cg2754578	1	2316653	MORN1	-0.17	4.65E-06	0.006827
cg2548856	6	1.17E+08	FAM26F	-0.16	4.80E-06	0.007015
cg0437678	2	2.19E+08	PNKD	-0.19	4.88E-06	0.007099
cg0184451	7	1.5E+08	ZNF862	-0.1	5.10E-06	0.007385
cg0327863	3	1.28E+08	NA	-0.18	5.44E-06	0.007841
cg0563850	15	1.02E+08	NA	-0.13	5.59E-06	0.007998
cg0880872	2	1.5E+08	KIF5C;MIR[illegible]	-0.15	5.60E-06	0.007998
cg1947565	14	1.01E+08	NA	-0.28	5.74E-06	0.008161
cg1586769	14	69438267	ACTN1	-0.11	5.79E-06	0.008195
cg0944710	12	15126020	PDE6H	-0.17	5.89E-06	0.008299
cg0107090	14	1.01E+08	NA	-0.44	6.15E-06	0.008627
cg2533249	11	64756382	BATF2	-0.12	6.43E-06	0.008979
cg0948947	11	1.02E+08	NA	-0.27	6.50E-06	0.009037
cg1402588	9	5436224	C9orf46	-0.17	6.58E-06	0.009108
cg2247825	6	36237982	PNPLA1	-0.14	6.73E-06	0.009275
cg2467876	15	74902976	CLK3	-0.14	6.81E-06	0.009344
cg0202602	17	79652703	HGS	-1.27	6.84E-06	0.009344
cg1455535	5	1.43E+08	ARHGAP26	-0.26	7.40E-06	0.010066

cg2093409	7	1117418	C7orf50	0.87	7.82E-06	0.010572
cg2267243	19	1854549	KLF16	-0.52	7.84E-06	0.010572
cg1671012	1	44716058	ERI3	-0.22	7.93E-06	0.010619
cg2489141	2	1.75E+08	WIPF1	1.94	7.96E-06	0.010619
cg0879134	10	13831250	FRMD4A	0.09	7.99E-06	0.010619
cg1117583	1	18806700	KLHDC7A	-0.12	8.01E-06	0.010619
cg1242570	4	75186779	NA	-0.12	8.06E-06	0.010641
cg0360359	8	29442834	NA	-0.37	8.20E-06	0.01078
cg1208230	1	6421246	ACOT7	-0.25	8.24E-06	0.010788
cg0083730	16	88473488	NA	-0.14	8.42E-06	0.010978
cg2331606	19	33761225	NA	-0.22	8.57E-06	0.011127
cg1794025	2	65179622	NA	-0.58	8.99E-06	0.011624
cg0500033	17	29817128	RAB11FIP4	-0.13	9.07E-06	0.01168
cg2335242	13	44566114	NA	-0.13	9.32E-06	0.011953
cg0011401	9	1.3E+08	SLC2A8	-0.13	9.76E-06	0.012466
cg2482367	19	5093491	KDM4B	-0.13	9.92E-06	0.012619
cg2191896	1	17751520	RCC2	-0.14	1.00E-05	0.01267
cg2226161	11	1.17E+08	BACE1	-0.42	1.02E-05	0.012769
cg1737853	17	41400661	NA	-0.16	1.02E-05	0.012769
cg2349892	20	47731131	STAU	-0.18	1.02E-05	0.012769
cg2635865	3	60740074	FHIT	-0.9	1.05E-05	0.013061
cg0636760	6	1.07E+08	NA	-0.34	1.06E-05	0.013061
cg0986250	1	1.81E+08	NA	-0.16	1.06E-05	0.013061
cg1377241	2	2.22E+08	EPHA4	0.13	1.06E-05	0.013061
cg1992614	10	661009	DIP2C	-0.65	1.07E-05	0.013133
cg0695896	10	45494806	C10orf25;Z	-0.71	1.08E-05	0.013204
cg0793780	19	3569036	NA	-0.31	1.09E-05	0.013275
cg0953552	16	88558065	ZFPM1	-0.12	1.11E-05	0.013414
cg0429927	17	25798741	KSR1	-0.13	1.11E-05	0.013414
cg0890689	20	34319899	RBM39	0.58	1.12E-05	0.013432
cg1219214	14	55849528	KIAA0831	-0.18	1.12E-05	0.013432
cg1977526	19	5288214	PTPRS	-0.11	1.14E-05	0.01362
cg1774952	17	42466567	ITGA2B	-0.23	1.15E-05	0.013687
cg0009368	9	1.4E+08	NDOR1	-0.2	1.16E-05	0.013702
cg1724298	1	2.44E+08	NA	-0.14	1.16E-05	0.013702
cg0933571	17	62255234	TEX2	-0.18	1.17E-05	0.013769
cg0178058	1	36348359	EIF2C1	-0.17	1.19E-05	0.013952
cg0806133	14	62068941	NA	-0.15	1.22E-05	0.014198
cg1961827	17	40715228	COASY	-0.17	1.22E-05	0.014198
cg2702820	6	1.66E+08	NA	1.66	1.23E-05	0.014261
cg2595331	12	1.12E+08	ACAD10	-0.19	1.30E-05	0.014962
cg0084415	12	6576111	VAMP1	-0.17	1.30E-05	0.014962
cg1138535	2	2.42E+08	AGXT	-0.19	1.31E-05	0.015022
cg1140902	1	44318844	ST3GAL3	-0.46	1.34E-05	0.01531
cg1348281	9	82183332	NA	-0.32	1.36E-05	0.015427
cg0191691	8	27461199	CLU	-0.21	1.36E-05	0.015427
cg1419511	9	1.15E+08	SUSD1	-0.15	1.38E-05	0.015597

cg2692269	6	33284542	ZBTB22	-0.15	1.40E-05	0.015767
cg2740570	21	43786824	TFF1	-0.17	1.41E-05	0.015823
cg2696470	12	1.26E+08	AACS	-0.16	1.48E-05	0.016549
cg0810526	17	56274480	EPX	-0.22	1.55E-05	0.01727
cg2611869	21	47599972	C21orf56	-0.13	1.57E-05	0.017431
cg0794808	10	9805340	NA	-0.22	1.59E-05	0.017591
cg1439265	17	6927424	BCL6B	-0.1	1.61E-05	0.01775
cg0434864	3	1.54E+08	NA	-0.11	1.67E-05	0.018347
cg0358624	7	1966361	MAD1L1	-0.05	1.74E-05	0.018983
cg0813468	1	38222311	EPHA10	-0.12	1.74E-05	0.018983
cg1848400	5	1.79E+08	MGAT4B	-0.49	1.77E-05	0.019178
cg1692023	17	80076378	CCDC57	-1.14	1.77E-05	0.019178
cg1976127	17	80232096	CSNK1D	-0.15	1.78E-05	0.01922
cg1546764	1	22141014	LDLRAD2	-2.31	1.79E-05	0.019261
cg1345860	9	1.31E+08	ENG	-0.17	1.80E-05	0.019303
cg1322179	13	48876919	RB1	-0.09	1.81E-05	0.019344
cg0673644	16	2801793	LOC10012[illegible]	-0.11	1.85E-05	0.019705
cg2100839	12	56120816	CD63	-0.15	1.87E-05	0.019783
cg1448053	17	55700565	MSI2	-0.11	1.87E-05	0.019783
cg0093735	6	86350540	SYNCRIP	-0.08	1.90E-05	0.020033
cg0846294	19	5288631	PTPRS	-0.13	1.91E-05	0.020071
cg0635025	1	87597369	LOC33952[illegible]	-0.42	1.92E-05	0.020109
cg0850015	5	1.5E+08	TCOF1	0.22	1.94E-05	0.020118
cg2678723	5	1.32E+08	IL4	-0.18	1.94E-05	0.020118
cg1175428	12	54610419	NA	-0.13	1.94E-05	0.020118
cg1241948	11	72933282	P2RY2	-0.12	2.03E-05	0.020947
cg1459303	17	56296980	MKS1	-0.11	2.04E-05	0.020947
cg0306015	3	48755856	IP6K2	-0.18	2.04E-05	0.020947
cg0414197	5	1.64E+08	NA	0.52	2.08E-05	0.021288
cg1467808	7	1.28E+08	SND1	-0.81	2.10E-05	0.021423
cg0034502	8	743875	NA	0.09	2.12E-05	0.021488
cg1596571	1	1.65E+08	PBX1	-0.17	2.12E-05	0.021488
cg0804135	17	25819112	KSR1	-0.15	2.14E-05	0.021621
cg1633589	12	1.24E+08	NA	-0.1	2.21E-05	0.022256
cg1716226	2	63276779	OTX1	-0.25	2.29E-05	0.022988
cg0600904	5	1.5E+08	ANXA6	-0.16	2.31E-05	0.023115
cg1050473	1	27226971	GPATCH3	-1.95	2.32E-05	0.023142
cg0773247	19	15390948	BRD4	-0.19	2.44E-05	0.024262
cg1979126	5	1.76E+08	HK3	-0.09	2.47E-05	0.024482
cg1889160	6	36319055	NA	-0.16	2.50E-05	0.024702
cg2157326	1	44482918	SLC6A9	0.16	2.55E-05	0.025117
cg0686415	7	1913918	MAD1L1	-0.27	2.56E-05	0.025137
cg1620537	6	33273460	TAPBP	-0.17	2.59E-05	0.025352
cg1040212	10	1.26E+08	NA	-0.21	2.60E-05	0.025371
cg0828733	19	1854633	KLF16	-0.79	2.62E-05	0.025487
cg2054569	8	48910778	NA	-0.09	2.63E-05	0.025505
cg0829541	5	1.57E+08	ADAM19	-0.4	2.70E-05	0.026103

cg2658498	12	93798975	NA	-0.64	2.73E-05	0.026232
cg2026385	1	8824177	RERE	-0.13	2.73E-05	0.026232
cg0004575	9	1.24E+08	PHF19	-0.51	2.78E-05	0.026565
cg1063506	2	1.06E+08	FHL2	-0.16	2.79E-05	0.026565
cg0872269	2	74378750	NA	-0.47	2.79E-05	0.026565
cg2301285	1	2.35E+08	NA	-0.34	2.81E-05	0.026674
cg1544270	5	1.77E+08	FGFR4	-0.52	2.82E-05	0.026689
cg1436044	9	1.33E+08	NA	-0.25	2.83E-05	0.026703
cg1887670	10	31107995	NA	-0.15	2.86E-05	0.026825
cg0572022	7	1.17E+08	ST7;ST7OT[illegible]	-0.13	2.86E-05	0.026825
cg2550000	10	31117548	NA	-0.09	2.88E-05	0.026852
cg1379164	10	1.2E+08	RAB11FIP2	-0.17	2.88E-05	0.026852
cg0181700	16	67978182	SLC12A4	-0.39	2.95E-05	0.027423
cg0642602	6	33232644	VPS52	-0.35	3.01E-05	0.027899
cg2641370	6	42306808	TRERF1	-0.11	3.05E-05	0.028186
cg2352570	14	70152683	KIAA0247	-0.1	3.08E-05	0.02838
cg2642382	17	25898825	KSR1	-0.34	3.09E-05	0.028389
cg1136144	11	68841024	TPCN2	-0.46	3.11E-05	0.028489
cg0829251	4	1.17E+08	NA	0.25	3.14E-05	0.028597
cg1127325	6	42716703	NA	-0.51	3.14E-05	0.028597
cg1513417	4	1.7E+08	SH3RF1	-0.2	3.16E-05	0.028696
cg0480221	13	24895902	C1QTNF9	0.54	3.18E-05	0.028795
cg2765551	11	2287421	NA	0.2	3.34E-05	0.03007
cg1830797	7	30953124	AQP1	-0.1	3.34E-05	0.03007
cg1865062	7	1914073	MAD1L1	-0.11	3.37E-05	0.030254
cg1476912	7	98722470	SMURF1	-0.19	3.39E-05	0.030347
cg1851005	10	77163383	C10orf41	0.07	3.44E-05	0.030707
cg0442091	19	39303993	LGALS4	-0.13	3.46E-05	0.030798
cg2270808	13	32780062	FRY	-0.45	3.61E-05	0.032042
cg0864804	1	11028561	C1orf127	-0.15	3.71E-05	0.032741
cg1127776	7	1.41E+08	TAS2R5	-0.35	3.72E-05	0.032741
cg0146448	11	67089352	LOC10013[illegible]	-0.19	3.72E-05	0.032741
cg1166454	22	30721636	TBC1D10A	-0.12	3.78E-05	0.033176
cg0187719	1	28559339	DNAJC8	-3.26	3.83E-05	0.033521
cg1606287	17	25798415	KSR1	-0.14	3.92E-05	0.034214
cg0472059	1	1.18E+08	FAM46C	0.11	3.95E-05	0.03438
cg0368990	2	1.36E+08	NA	0.64	4.06E-05	0.03524
cg2275869	10	51572216	NCOA4	-0.82	4.08E-05	0.035316
cg2290022	2	25391773	POMC	-0.18	4.19E-05	0.036143
cg0492374	1	2.12E+08	RD3	-0.11	4.20E-05	0.036143
cg1832871	1	7727380	CAMTA1	-0.11	4.21E-05	0.036143
cg1461125	17	17946468	C17orf39	-0.28	4.26E-05	0.036458
cg1342428	1	26624129	UBXN11	-0.27	4.27E-05	0.036458
cg1002793	22	39799092	MAP3K7IP[illegible]	-0.23	4.33E-05	0.03687
cg2226511	17	4871194	SPAG7	-1.74	4.37E-05	0.03701
cg1404352	11	64642516	EHD1	-0.12	4.37E-05	0.03701
cg0101208	8	71025759	NCOA2	-0.1	4.40E-05	0.037164

cg1420918	4	38980960	TMEM156	-0.2	4.46E-05	0.037554
cg1594626	7	95858349	SLC25A13	0.64	4.47E-05	0.037554
cg2092371	18	32557027	MAPRE2	-1.17	4.54E-05	0.03804
cg0779737	12	44316362	TMEM117	0.63	4.62E-05	0.038607
cg0293095	20	48806066	CEBPB	-0.12	4.67E-05	0.038819
cg0101926	1	1.55E+08	NA	-0.13	4.67E-05	0.038819
cg2687313	11	9191490	DENND5A	-0.35	4.70E-05	0.038863
cg0369587	11	65547172	DKFZp761E	-0.06	4.70E-05	0.038863
cg1139658	6	33273477	TAPBP	-0.18	4.83E-05	0.039833
cg1435548	21	22373039	NCAM2	-0.12	5.08E-05	0.041785
cg1395100	1	1.47E+08	LOC72898[illegible]	-0.12	5.10E-05	0.04184
cg2167275	2	48340131	NA	-0.42	5.23E-05	0.042794
cg2252529	19	50249464	TSKS	0.09	5.32E-05	0.043418
cg0426058	2	1E+08	REV1;REV1	-0.19	5.53E-05	0.045015
cg0247328	2	9752386	YWHAQ	-0.11	5.55E-05	0.045061
cg1545682	11	61717225	BEST1	-0.13	5.57E-05	0.045107
cg0333560	7	1.39E+08	HIPK2	-0.18	5.61E-05	0.045278
cg1197025	13	42188629	KIAA0564	-0.34	5.62E-05	0.045278
cg1045603	17	76169212	NA	-0.29	5.77E-05	0.046368
cg1809235	14	77242220	VASH1	0.37	5.97E-05	0.047853
cg0714803	6	32061160	TNXB	-0.24	6.01E-05	0.048051
cg2303827	1	2.27E+08	ITPKB	-0.07	6.08E-05	0.048487
cg0393595	16	23511855	GGA2	-0.09	6.26E-05	0.049796

Model adjusted for maternal [age at enrollment (continuous), smoking status (smoking during pregnancy / former / never), college graduate (yes / no), maternal atopy history (yes / no)], children [child’s sex (female / male), race (white / black / other), gestational age (continuous), age at blood drawn (continuous)], and the first five ReFACTor components.

Table S5. Stratified analysis of methylation sites identified in the cord blood DNA methylation and mid-childhood IgE analysis. Results are expressed as the change in log(IgE) concentration per 1% increase in cord blood methylation value.

CpG	CHR	MAPINFO	Gene	White Estimate	P value	Non-white Estimate	P value	Female Estimate	P value	Male Estimate	P value
cg06226630	4	48493420	ZAR1	-0.47	5.81E-09	-0.07	7.53E-01	-0.38	3.59E-04	-0.35	1.64E-02
cg03307893	15	26108683	ATP10A	-0.67	9.63E-05	-1.21	7.47E-06	-0.51	1.52E-02	-0.90	8.20E-08
cg13322072	7	98784083	KPNA7	-0.08	1.98E-01	-0.12	1.40E-04	-0.15	6.98E-05	-0.10	5.34E-04
cg16797808	14	99948289	SETD3;CCNK	-0.72	8.14E-04	-1.26	4.22E-05	-1.33	1.49E-12	-0.46	4.45E-02
cg09535168	19	19431582	MAU2	-1.43	7.11E-05	-1.59	1.78E-02	-1.50	2.52E-04	-1.55	7.22E-04
cg04122974	22	39916495	ATF4	-2.80	9.12E-06	-3.62	5.69E-02	-3.56	3.45E-05	-2.32	4.69E-03
cg01527777	11	71956145	PHOX2A	-0.26	1.79E-05	-0.27	1.18E-03	-0.28	1.15E-04	-0.22	7.22E-04
cg24575275	7	1094737	C7orf50	-0.23	5.58E-03	-0.42	1.63E-05	-0.43	4.43E-06	-0.17	4.11E-02
cg14920426	5	176924420	PDLIM7	-0.94	2.63E-03	-1.16	3.20E-03	-1.23	2.30E-03	-0.64	1.97E-02
cg05399209	19	46010836	VASP	-1.20	7.87E-04	-1.29	3.15E-03	-1.20	9.61E-04	-1.35	9.06E-05
cg09507928	5	140027484	IK;NDUFA2	-1.90	1.02E-03	-2.21	8.86E-06	-2.19	5.60E-03	-1.76	1.91E-03
cg24114890	14	23834349	EFS	-0.91	9.23E-08	-0.33	3.75E-01	-0.42	1.31E-01	-0.95	2.82E-05
cg05063806	1	36772417	C1orf113	-1.06	1.24E-03	-1.29	4.75E-03	-1.14	5.55E-03	-1.06	4.78E-03
cg14167858	1	120199593	--	0.11	5.42E-05	0.05	3.37E-01	0.07	1.23E-01	0.11	4.15E-06
cg02228675	17	40259724	DHX58	-0.27	1.20E-05	-0.40	4.35E-03	-0.40	1.22E-04	-0.17	5.80E-03
cg14607755	9	139962279	C9orf140	-0.52	5.93E-04	-0.43	2.57E-02	-0.61	3.70E-04	-0.34	4.58E-02
cg24630419	1	217311608	ESRRG	-0.44	4.74E-04	-0.45	1.40E-03	-0.44	1.36E-02	-0.36	7.64E-04
cg27212903	15	92708880	SLCO3A1	1.12	4.25E-06	0.91	7.78E-02	1.10	1.35E-05	0.87	1.04E-02
cg14605590	9	94900583	LOC100128076	-0.17	8.78E-05	-0.13	4.11E-02	-0.16	6.85E-03	-0.12	9.87E-03

Model adjusted for maternal [age at enrollment (continuous), smoking status (smoking during pregnancy / former / never), college graduate (yes / no), maternal atopy history (yes / no)], children [gestational age (continuous), age at blood drawn (continuous)], and the first five ReFACTor components. We further adjusted for child's sex when we modeled white vs. non-white subpopulation; and adjusted for child's race when we modeled female vs. male subpopulation.

Corresponding model: $\log(IgE) = \alpha_0 + \alpha_1 CpG_{\_cbi} + \alpha_2 X_2 + \ldots + \alpha_p X_p + \varepsilon$

Table S6. Stratified analysis of methylation sites identified in the life course analysis. For the prenatal analysis, results are expressed as the change in log(IgE) concentration per 1% increase in cord blood methylation value. While for the postnatal analysis, results are expressed as the change in log(IgE) concentration per 1% increase in Δ-DNAm methylation value.

Life course analysis—prenatal influence											
				**White**		**Non-white**		**Female**		**Male**	
**CpG**	**CHR**	**MAPINFO**	**Gene**	**Estimate**	**P value**	**Estimate**	**P value**	**Estimate**	**P value**	**Estimate**	**P value**
cg25854298	10	73936754	ASCC1	-0.36	3.87E-06	-0.36	8.02E-09	-0.42	4.44E-04	-0.36	6.95E-06
cg16416603	20	57593014	TUBB1	0.52	4.55E-05	0.76	7.46E-05	0.68	7.38E-04	0.49	2.05E-04
cg11848324	14	20403845	OR4K1	0.70	4.09E-04	0.41	8.29E-03	0.69	2.33E-04	0.30	1.25E-01
cg19549714	18	3447713	TGIF1	-2.04	1.23E-05	-2.00	2.40E-07	-2.18	1.10E-04	-1.60	5.87E-03
cg23933289	1	178998656	FAM20B	-0.16	1.22E-03	-0.18	2.66E-07	-0.32	8.17E-05	-0.16	3.02E-04
cg24576940	7	150648283	KCNH2	-1.46	3.73E-04	-1.54	3.31E-07	-1.74	3.84E-09	-1.37	1.88E-03
cg13443997	9	139743586	PHPT1	-3.57	1.91E-05	-2.66	4.21E-02	-3.24	1.70E-02	-2.97	2.08E-03
cg19954205	12	122211282	TMEM120B	-1.45	1.18E-05	-1.20	7.20E-07	-1.43	1.15E-03	-1.02	4.21E-03
cg01782059	1	44715942	ERI3	-1.10	1.25E-04	-1.17	3.22E-04	-0.78	1.84E-01	-1.20	1.82E-07
cg02133716	8	128981622	PVT1	-0.51	2.50E-04	-0.46	1.68E-02	-0.66	8.77E-08	-0.24	7.52E-02
cg06226630	4	48493420	ZAR1	-0.45	1.42E-07	-0.07	7.95E-01	-0.41	1.97E-05	-0.24	1.75E-01
cg20675173	3	43795541	--	-1.37	1.35E-05	-1.22	1.03E-06	-1.72	3.32E-03	-1.01	2.28E-03
cg08067346	16	25011481	ARHGAP17	-0.25	5.17E-04	-0.24	1.44E-02	-0.29	1.04E-03	-0.25	1.33E-04
cg09597192	6	32141591	AGPAT1	-0.26	1.36E-02	-0.36	1.00E-05	-0.47	1.46E-08	-0.21	3.99E-03
cg16096766	13	52419714	FLJ37307	-0.49	1.23E-05	-0.11	4.59E-01	-0.61	1.38E-05	-0.29	1.45E-02
cg00026222	1	2144244	--	-1.15	2.75E-05	-0.71	1.29E-01	-0.88	1.10E-02	-0.84	9.33E-03
cg24575275	7	1094737	C7orf50	-0.24	4.66E-03	-0.31	2.49E-06	-0.47	5.39E-06	-0.16	5.96E-02

Life course analysis—postnatal influences (top 20 methylation sites)											
				**White**		**Non-white**		**Female**		**Male**	
**CpG**	**CHR**	**MAPINFO**	**Gene**	**Estimate**	**P value**	**Estimate**	**P value**	**Estimate**	**P value**	**Estimate**	**P value**
cg11699125	1	6341327	ACOT7	-0.27	5.10E-17	-0.23	7.76E-11	-0.28	2.61E-12	-0.23	7.38E-16
cg02970679	17	56269818	EPX	-0.29	8.79E-18	-0.25	3.97E-12	-0.28	1.04E-18	-0.24	4.32E-16
cg24491618	7	150649807	KCNH2	-0.20	2.13E-11	-0.19	2.12E-16	-0.22	1.94E-16	-0.14	2.42E-04
cg01614759	10	45495435	C10orf25;ZNF22	-0.30	1.81E-09	-0.31	1.67E-11	-0.34	1.55E-14	-0.24	4.93E-13
cg13054523	17	81055722	--	-0.49	3.39E-11	-0.36	6.06E-07	-0.45	2.93E-09	-0.46	3.88E-15
cg21220721	1	6341230	ACOT7	-0.10	5.47E-07	-0.11	1.30E-14	-0.12	4.40E-10	-0.09	5.01E-06
cg10065736	12	117440120	FBXW8	-0.18	7.34E-10	-0.18	5.14E-07	-0.22	6.32E-19	-0.15	4.76E-08
cg06558622	7	149543177	ZNF862	-0.42	2.03E-11	-0.47	3.56E-10	-0.39	1.02E-04	-0.41	9.23E-11
cg25854298	10	73936754	ASCC1	-0.44	8.33E-09	-0.40	1.80E-13	-0.43	2.88E-05	-0.38	1.74E-07
cg05300717	11	65546210	DKFZp761E198	-0.37	8.58E-11	-0.35	2.86E-09	-0.38	1.28E-11	-0.30	1.05E-07
cg09596645	3	181897670	--	-0.28	2.52E-09	-0.20	1.34E-02	-0.31	1.06E-07	-0.21	2.57E-06
cg19928703	13	30143971	SLC7A1	-0.38	4.00E-08	-0.31	1.06E-12	-0.36	1.17E-08	-0.32	5.03E-06

cg18368116	14	21436271	--	-0.30	1.75E-08	-0.27	1.91E-12	-0.32	1.11E-13	-0.25	3.23E-09
cg20885063	17	17939419	ATPAF2	-0.23	2.37E-08	-0.23	4.05E-12	-0.24	1.64E-12	-0.16	2.97E-03
cg08077807	14	62001072	PRKCH	-0.87	2.75E-08	-0.77	4.49E-05	-0.74	1.13E-04	-0.74	1.26E-08
cg07908654	13	41631052	--	-0.25	3.86E-09	-0.26	1.51E-03	-0.35	1.84E-13	-0.20	5.29E-06
cg08940169	16	88540241	ZFPM1	-0.18	3.50E-09	-0.20	4.44E-06	-0.21	9.50E-08	-0.17	2.39E-08
cg18879389	21	43771120	TFF2	-0.29	7.61E-11	-0.28	6.73E-12	-0.35	9.42E-11	-0.24	1.64E-04
cg20263733	3	130616293	ATP2C1	-0.54	5.28E-10	-0.50	7.45E-12	-0.62	2.45E-06	-0.49	3.00E-05
cg02985445	7	97908505	--	-0.18	5.10E-08	-0.23	1.07E-06	-0.21	1.74E-07	-0.16	3.72E-06

Life course analysis—prenatal and postnatal influence—prenatal influence											
				**White**		**Non-white**		**Female**		**Male**	
**CpG**	**CHR**	**MAPINFO**	**Gene**	**Estimate**	**P value**	**Estimate**	**P value**	**Estimate**	**P value**	**Estimate**	**P value**
cg25854298	10	73936754	ASCC1	-0.36	3.87E-06	-0.36	8.02E-09	-0.42	4.44E-04	-0.36	6.95E-06
cg11848324	14	20403845	OR4K1	0.70	4.09E-04	0.41	8.29E-03	0.69	2.33E-04	0.30	1.25E-01
cg23933289	1	178998656	FAM20B	-0.16	1.22E-03	-0.18	2.66E-07	-0.32	8.17E-05	-0.16	3.02E-04
cg24576940	7	150648283	KCNH2	-1.46	3.73E-04	-1.54	3.31E-07	-1.74	3.84E-09	-1.37	1.88E-03
cg02133716	8	128981622	PVT1	-0.51	2.50E-04	-0.46	1.68E-02	-0.66	8.77E-08	-0.24	7.52E-02
cg08067346	16	25011481	ARHGAP17	-0.25	5.17E-04	-0.24	1.44E-02	-0.29	1.04E-03	-0.25	1.33E-04
cg09597192	6	32141591	AGPAT1	-0.26	1.36E-02	-0.36	1.00E-05	-0.47	1.46E-08	-0.21	3.99E-03

Life course analysis— prenatal and postnatal influence—postnatal influence											
				**White**		**Non-white**		**Female**		**Male**	
**CpG**	**CHR**	**MAPINFO**	**Gene**	**Estimate**	**P value**	**Estimate**	**P value**	**Estimate**	**P value**	**Estimate**	**P value**
cg25854298	10	73936754	ASCC1	-0.44	8.33E-09	-0.40	1.80E-13	-0.43	2.88E-05	-0.38	1.74E-07
cg11848324	14	20403845	OR4K1	0.48	3.36E-04	0.50	7.40E-04	0.34	9.25E-02	0.29	9.20E-02
cg23933289	1	178998656	FAM20B	-0.20	7.78E-06	-0.17	8.01E-08	-0.24	7.34E-04	-0.14	3.54E-05
cg24576940	7	150648283	KCNH2	-1.64	8.58E-05	-1.66	2.95E-08	-1.89	1.71E-11	-1.42	2.19E-03
cg02133716	8	128981622	PVT1	-0.62	8.25E-09	-0.46	5.26E-06	-0.65	1.64E-12	-0.42	1.26E-04
cg08067346	16	25011481	ARHGAP17	-0.34	2.19E-07	-0.26	5.14E-05	-0.33	9.07E-08	-0.32	1.35E-05
cg09597192	6	32141591	AGPAT1	-0.24	2.34E-03	-0.32	5.52E-10	-0.40	2.99E-12	-0.18	5.37E-04

Model adjusted for maternal [age at enrollment (continuous), smoking status (smoking during pregnancy / former / never), college graduate (yes / no), maternal atopy history (yes / no)], children [gestational age (continuous), age at blood drawn (continuous)], and the first five ReFACTor components. We further adjusted for child's sex when we modeled white vs. non-white subpopulation; and adjusted for child's race when we modeled female vs. male subpopulation.

Corresponding model: $\log(IgE) = \beta_0 + \beta_1 CpG\_{_{cbi}} + \beta_2 \Delta CpG_i + \beta_3 X_3 + \ldots + \beta_p X_p + \eta$

Life course analysis—prenatal influence: association between cord blood methylation and mid-childhood IgE independent of postnatal changes in DNA methylation; which correspond to $\beta_1 CpG\_{_{cbi}}$;

Life course analysis—postnatal influence: association between changes in postnatal DNA methylation from birth and mid-childhood IgE independent of baseline / cord blood DNA methylation; which correspond to ΔCpG_i

Table S7. Association between cord blood DNA methylation and mid-childhood IgE (FDR threshold <0.05)—we estimated cell proportions using blood reference panels for cord blood. Results are expressed as the change in log(IgE) concentration per 1% increase in cord blood methylation value.

CpG	CHR	MAPINFO	Gene	Estimate	P value
cg06226630	4	48493420	ZAR1	-0.38	2.23E-06
cg03307893	15	26108683	ATP10A	-0.58	7.14E-05
cg13322072	7	98784083	KPNA7	-0.09	4.05E-04
cg16797808	14	99948289	SETD3;CCNK	-0.84	3.25E-06
cg09535168	19	19431582	MAU2	-1.34	1.92E-06
cg04122974	22	39916495	ATF4	-2.66	7.07E-06
cg01527777	11	71956145	PHOX2A	-0.22	2.01E-05
cg24575275	7	1094737	C7orf50	-0.27	5.73E-05
cg14920426	5	176924420	PDLIM7	-0.83	1.95E-05
cg05399209	19	46010836	VASP	-1.07	2.83E-05
cg09507928	5	140027484	IK;NDUFA2	-1.76	8.08E-07
cg24114890	14	23834349	EFS	-0.69	2.20E-06
cg05063806	1	36772417	C1orf113	-1.05	2.58E-05
cg14167858	1	120199593	--	0.01	0.491598
cg02228675	17	40259724	DHX58	-0.27	6.06E-06
cg14607755	9	139962279	C9orf140	-0.45	5.72E-06
cg24630419	1	217311608	ESRRG	-0.36	5.85E-06
cg27212903	15	92708880	SLCO3A1	0.79	2.55E-05
cg14605590	9	94900583	LOC100128076	-0.13	1.76E-05

Model adjusted for maternal [age at enrollment (continuous), smoking status (smoking during pregnancy / former / never), college graduate (yes / no), maternal atopy history (yes / no)], children [child's sex (female / male), race/ethnicity (white / black / other), gestational age (continuous), age at blood drawn (continuous)], and estimated cell proportions (percentage of monocyte, percentage of CD8T cell, percentage of CD4T cell, percentage of NK cell, percentage of B cell, percentage of nucleated red blood cells).

Table S8. Association between cord blood DNA methylation and mid-childhood IgE (FDR threshold <0.05) among the 217 children who had DNA methylation measurements both at birth and in mid-childhood. Results are expressed as the change in log(IgE) concentration per 1% increase in cord blood methylation value.

CpG	CHR	MAPINFO	Gene	Estimate	P value
cg06226630	4	48493420	ZAR1	-0.39	7.59E-07
cg03307893	15	26108683	ATP10A	-0.68	1.20E-05
cg13322072	7	98784083	KPNA7	-0.12	1.25E-09
cg16797808	14	99948289	SETD3;CCNK	-0.72	7.55E-05
cg09535168	19	19431582	MAU2	-1.49	6.38E-07
cg04122974	22	39916495	ATF4	-2.69	5.97E-06
cg01527777	11	71956145	PHOX2A	-0.23	1.99E-07
cg24575275	7	1094737	C7orf50	-0.29	1.26E-06
cg14920426	5	176924420	PDLIM7	-0.90	3.90E-05
cg05399209	19	46010836	VASP	-1.23	5.71E-06
cg09507928	5	140027484	IK;NDUFA2	-1.73	2.65E-05
cg24114890	14	23834349	EFS	-0.71	3.27E-05
cg05063806	1	36772417	C1orf113	-1.20	2.44E-05
cg14167858	1	120199593	--	0.09	1.97E-05
cg02228675	17	40259724	DHX58	-0.27	2.96E-05
cg14607755	9	139962279	C9orf140	-0.47	6.87E-06
cg24630419	1	217311608	ESRRG	-0.36	2.45E-05
cg27212903	15	92708880	SLCO3A1	0.95	4.92E-06
cg14605590	9	94900583	LOC100128076	-0.14	1.81E-04

Model adjusted for maternal [age at enrollment (continuous), smoking status (smoking during pregnancy / former / never), college graduate (yes / no), maternal atopy history (yes / no)], children [child's sex (female / male), race/ethnicity (white / black / other), gestational age (continuous), age at blood drawn (continuous)], and the cell-type proxys using the first five ReFACTor components estimated from cord blood.

Table S9. Genetic infuences on DNA methylation of selected top hits—results from mQTL from an independent cohort. We restricted to SNPs with MAF >5% and within 500Kb away from the methylation site.

Life course analysis—"early programming"							
				**Birth**		**Childhood**	
**CpG**	**CHR**	**MAPINFO**	**Gene**	**cis**	**No. of SNP**	**cis**	**No. of SNP**
cg25854298	10	73936754	ASCC1	--	--	--	--
cg16416603	20	57593014	TUBB1	--	--	--	--
cg11848324	14	20403845	OR4K1	--	--	--	--
cg19549714	18	3447713	TGIF1	--	--	Yes	2
cg23933289	1	178998656	FAM20B	--	--	--	--
cg24576940	7	150648283	KCNH2	Yes	29	Yes	79
cg13443997	9	139743586	PHPT1	--	--	--	--
cg19954205	12	122211282	TMEM120B	--	--	--	--
cg01782059	1	44715942	ERI3	--	--	--	--
cg02133716	8	128981622	PVT1	--	--	--	--
cg06226630	4	48493420	ZAR1	--	--	--	--
cg20675173	3	43795541	--	--	--	--	--
cg08067346	16	25011481	ARHGAP17	--	--	--	--
cg09597192	6	32141591	AGPAT1	--	--	--	--
cg16096766	13	52419714	FLJ37307	--	--	--	--
cg00026222	1	2144244	--	--	--	--	--

Life course analysis—postnatal influences (top 20 methylation sites)							
				**Birth**		**Childhood**	
**CpG**	**CHR**	**MAPINFO**	**Gene**	**cis**	**No. of SNP**	**cis**	**No. of SNP**
cg11699125	1	6341327	ACOT7	--	--	--	--
cg02970679	17	56269818	EPX	--	--	--	--
cg24491618	7	150649807	KCNH2	--	--	--	--
cg01614759	10	45495435	C10orf25;ZNF22	--	--	--	--
cg13054523	17	81055722	--	--	--	--	--
cg21220721	1	6341230	ACOT7	--	--	--	--
cg10065736	12	117440120	FBXW8	--	--	--	--
cg06558622	7	149543177	ZNF862	--	--	--	--
cg25854298	10	73936754	ASCC1	--	--	--	--
cg05300717	11	65546210	DKFZp761E198	--	--	--	--
cg09596645	3	181897670	--	--	--	--	--
cg19928703	13	30143971	SLC7A1	--	--	--	--
cg18368116	14	21436271	--	--	--	--	--
cg20885063	17	17939419	ATPAF2	--	--	--	--

cg08077807	14	62001072	PRKCH	--	--	--	--
cg07908654	13	41631052	--	--	--	--	--
cg08940169	16	88540241	ZFPM1	--	--	--	--
cg18879389	21	43771120	TFF2	Yes	6	Yes	2
cg20263733	3	130616293	ATP2C1	--	--	--	--
cg02985445	7	97908505	--	--	--	--	--

Life course analysis—"early programming "and postnatal influences

CpG	CHR	MAPINFO	Gene	Birth cis	No. of SNP	Childhood cis	No. of SNP
cg25854298	10	73936754	ASCC1	--	--	--	--
cg11848324	14	20403845	OR4K1	--	--	--	--
cg23933289	1	178998656	FAM20B	--	--	--	--
cg24576940	7	150648283	KCNH2	Yes	29	Yes	79
cg02133716	8	128981622	PVT1	--	--	--	--
cg08067346	16	25011481	ARHGAP17	--	--	--	--
cg09597192	6	32141591	AGPAT1	--	--	--	--

Life course analysis—postnatal influences (asthma pathway)

CpG	CHR	MAPINFO	Gene	Birth cis	No. of SNP	Childhood cis	No. of SNP
cg26787239	5	132008525	IL4	--	--	--	--
cg01310029	3	3152374	IL5RA	--	--	--	--
cg02970679	17	56269818	EPX	--	--	--	--
cg25173129	17	56269410	EPX	--	--	--	--
cg27469152	17	56282313	EPX	--	--	--	--
cg18421167	17	56276490	EPX	--	--	--	--
cg08105265	17	56274480	EPX	--	--	--	--
cg12819873	11	57157632	PRG2	--	--	--	--
cg15700636	11	57156050	PRG2	--	--	--	--
cg08295410	5	156990663	ADAM19	--	--	--	--

Table S10. Summary statistics of selected methylation sites and their sign of associations for the prenatal and postnatal influences.

CpG	CHR	MAPINFO	Gene	Cord blood	Mid-childhood	ΔCpG	Sign of prenatal influences	Sign of postnatal influences
cg25854298	10	73936754	ASCC1	92.78% (5.48%)	97.20% (2.13%)	+4.41%	-	-
cg11848324	14	20403845	OR4K1	96.92% (1.52%)	97.38% (1.27%)	+0.46%	+	+
cg23933289	1	178998656	FAM20B	90.23% (5.32%)	92.64% (3.21%)	+2.41%	-	-
cg24576940	7	150648283	KCNH2	95.64% (3.66%)	98.37 (0.87%)	+2.72%	-	-
cg02133716	8	128981622	PVT1	93.55% (5.18%)	97.23% (1.67%)	+3.68%	-	-
cg08067346	16	25011481	ARHGAP17	92.60% (5.73%)	95.95% (3.19%)	+4.41%	-	-
cg09597192	6	32141591	AGPAT1	93.58% (3.62%)	95.20% (2.61%)	+1.62%	-	-

Table S11. Association between mid-childhood peripheral blood DNA methylation and mid-childhood IgE (FDR threshold <0.05) among the 210 children who had DNA methylation measurements both at mid-childhood only (top 20 methylation sites). Results are expressed as the change in log(IgE) concentration per 1% increase in mid-childhood methylation value.

				***Concurrent analysis* (N=243)**		
**CpG**	**CHR**	**MAPINFO**	**Gene**	**Estimate**	**P value**	**FDR**
cg11699125	1	6341327	ACOT7	-0.21	3.51E-09	1.38E-05
cg02970679	17	56269818	EPX	-0.23	2.91E-10	2.18E-06
cg24491618	7	150649807	KCNH2	-0.19	5.44E-14	2.85E-09
cg01614759	10	45495435	C10orf25	-0.19	9.80E-08	1.58E-04
cg13054523	17	81055722	--	-0.30	1.85E-08	4.80E-05
cg21220721	1	6341230	ACOT7	-0.11	2.98E-14	2.34E-09
cg10065736	12	117440120	FBXW8	-0.17	6.80E-10	3.96E-06
cg06558622	7	149543177	ZNF862	-0.24	6.07E-06	3.79E-03
cg25854298	10	73936754	ASCC1	-0.26	1.06E-08	3.14E-05
cg05300717	11	65546210	DKFZp761E198	-0.34	3.45E-08	7.19E-05
cg09596645	3	181897670	--	-0.18	6.81E-07	6.73E-04
cg19928703	13	30143971	SLC7A1	-0.23	7.73E-07	7.50E-04
cg18368116	14	21436271	--	-0.21	1.22E-08	3.45E-05
cg20885063	17	17939419	ATPAF2	-0.19	8.87E-08	1.48E-04
cg08077807	14	62001072	PRKCH	-0.36	1.35E-02	5.33E-01
cg07908654	13	41631052	--	-0.15	3.42E-03	2.81E-01
cg08940169	16	88540241	ZFPM1	-0.19	6.89E-09	2.28E-05
cg18879389	21	43771120	TFF2	-0.22	3.37E-04	6.95E-02
cg20263733	3	130616293	ATP2C1	-0.29	3.04E-07	3.81E-04
cg02985445	7	97908505	--	-0.21	5.58E-12	1.03E-07

Model adjusted for maternal [age at enrollment (continuous), smoking status (smoking during pregnancy / former / never), college graduate (yes / no), maternal atopy history (yes / no)], children [child's sex (female / male), race/ethnicity (white / black / other), gestational age (continuous), age at blood drawn (continuous)], and the cell-type proxys using the first five ReFACTor components estimated from cord blood.
