# Supplementary material for: Epigenome-wide association study of total serum immunoglobulin E in children: a life course approach
Source: Clin Epigenetics. 2018 Apr 17;10:55. doi: 10.1186/s13148-018-0488-x (PMC5905182; doi:10.1186/s13148-018-0488-x)

Figure S1. Scatter plot (cord blood DNA methylation and mid-childhood total serum IgE).

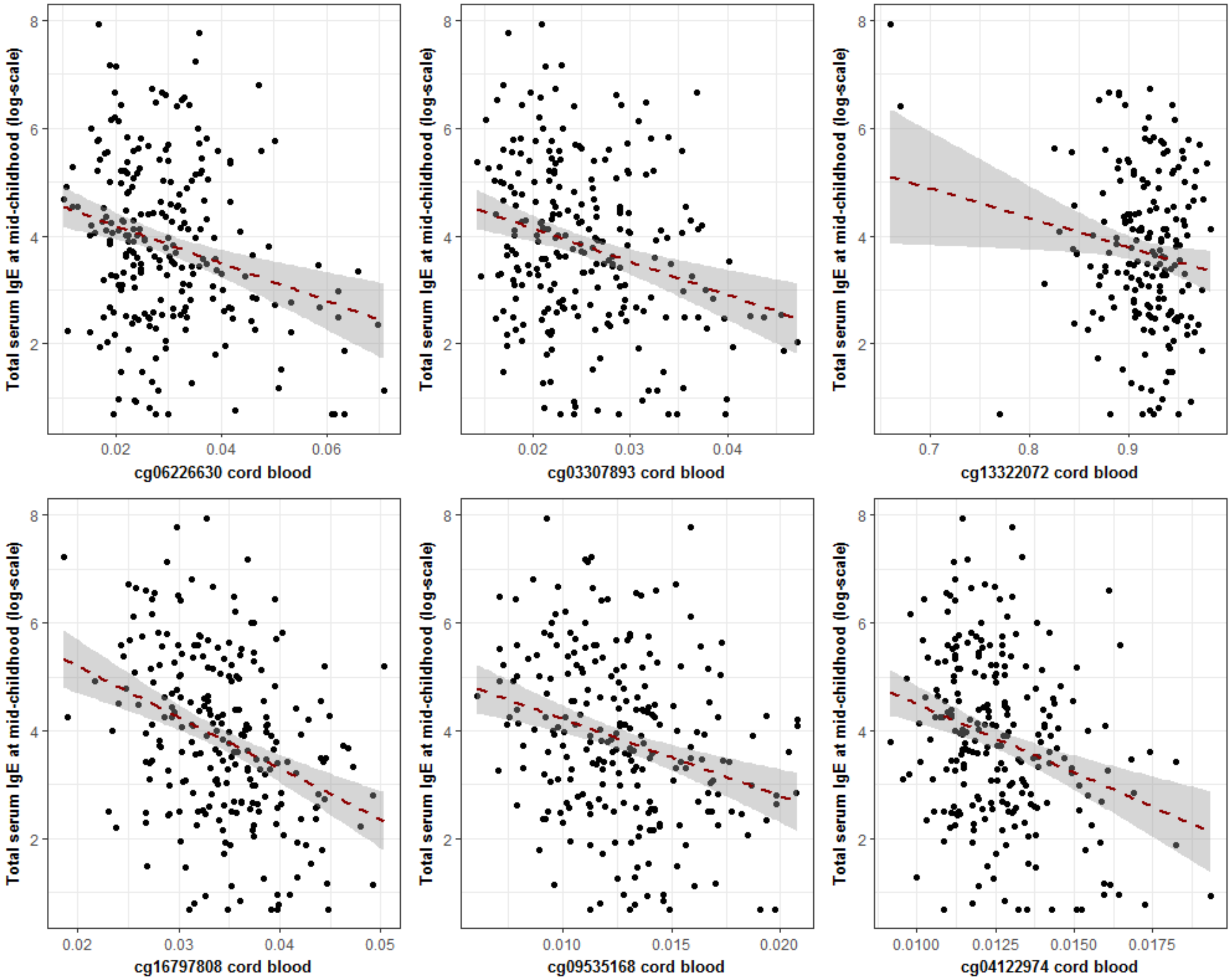

Figure S1 continue. Scatter plot (life course analysis: prenatal influence—top 6 methylation sites).

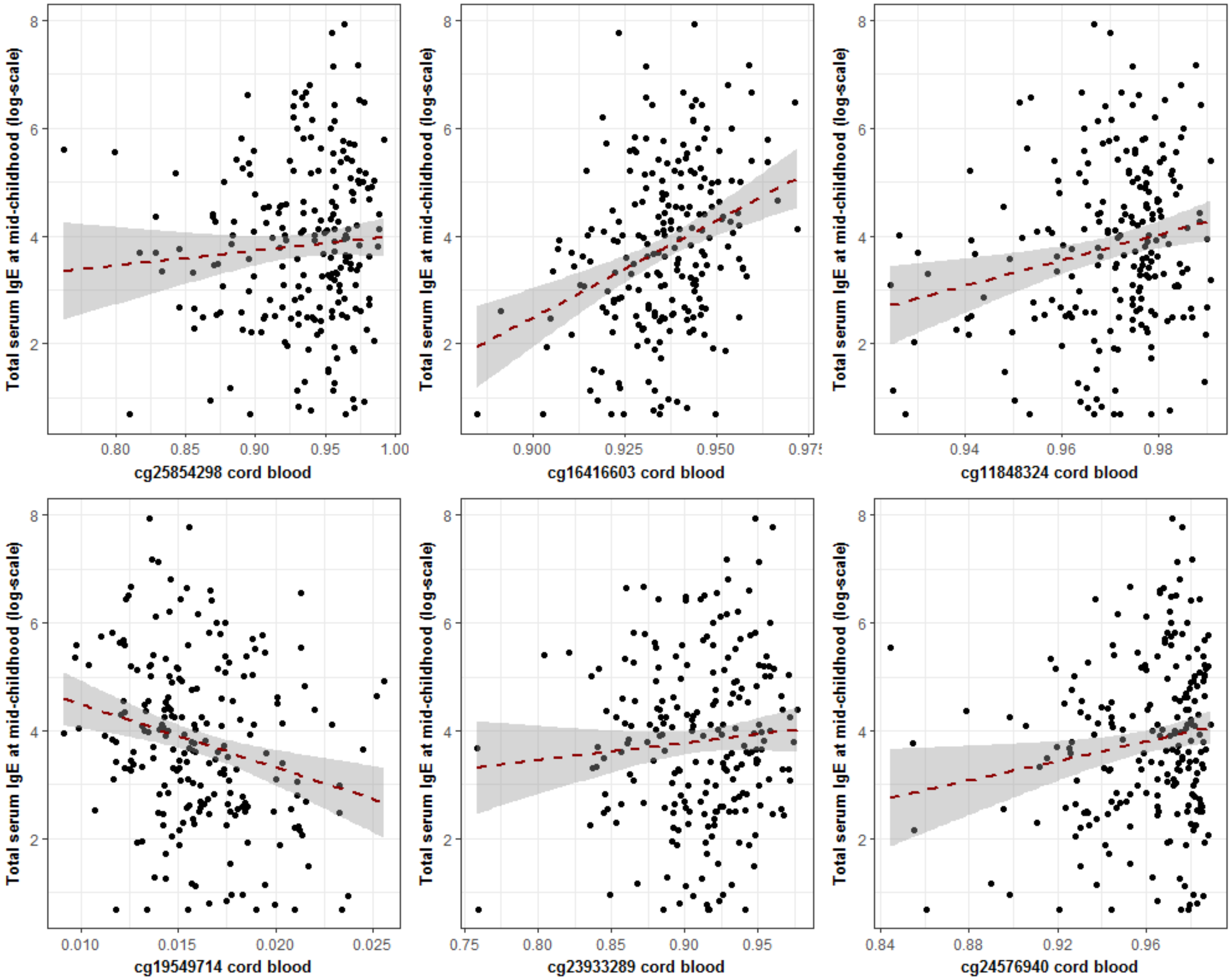

Figure S1 continue. Scatter plot (life course analysis: postnatal influences—top 6 methylation sites).

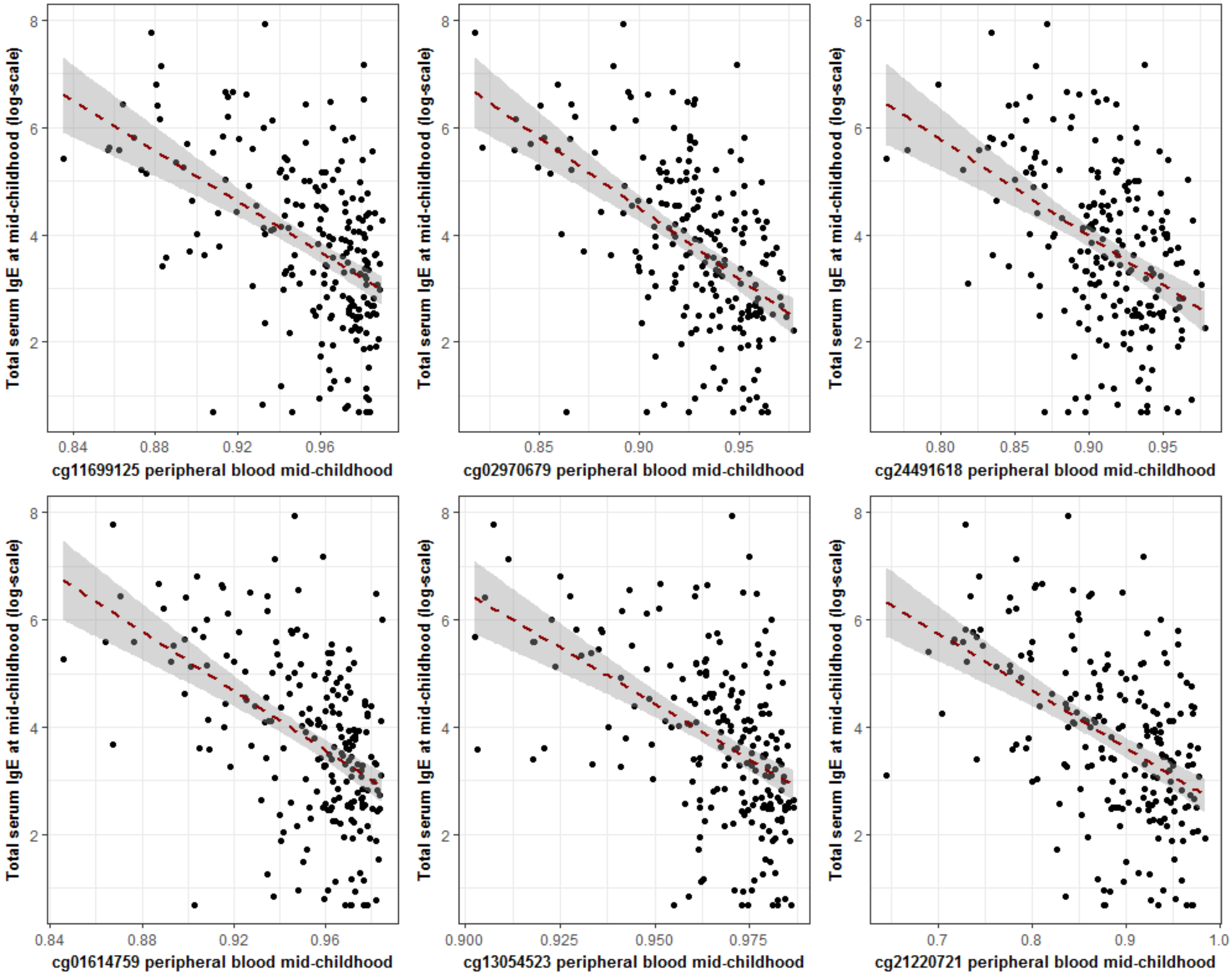

Figure S1 continue. Scatter plot (life course analysis: postnatal influences (asthma pathway)—top 6 methylation sites).

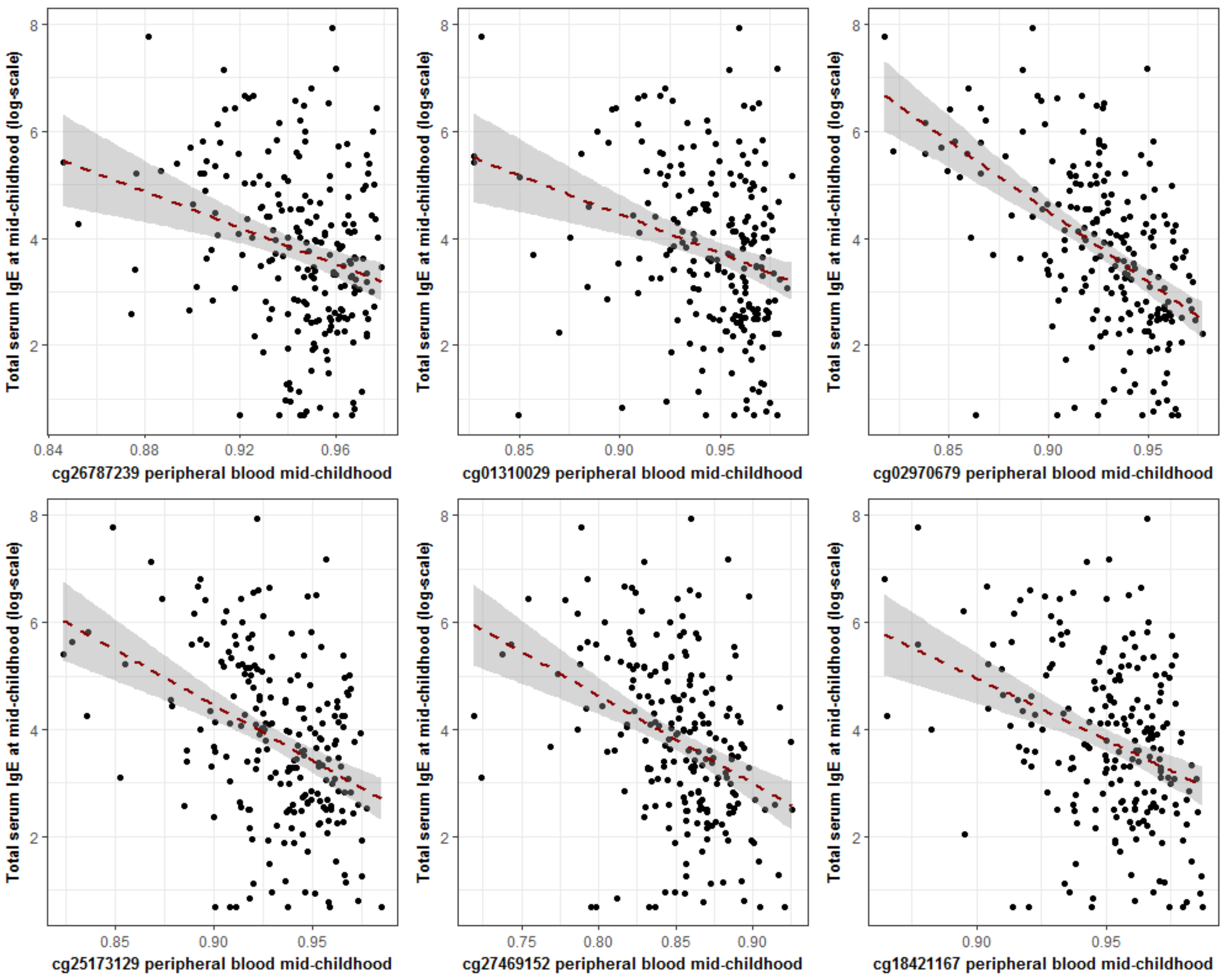

Figure S1 continue. Scatter plot (life course analysis—contribution of prenatal and postnatal influences—top 6 methylation sites).

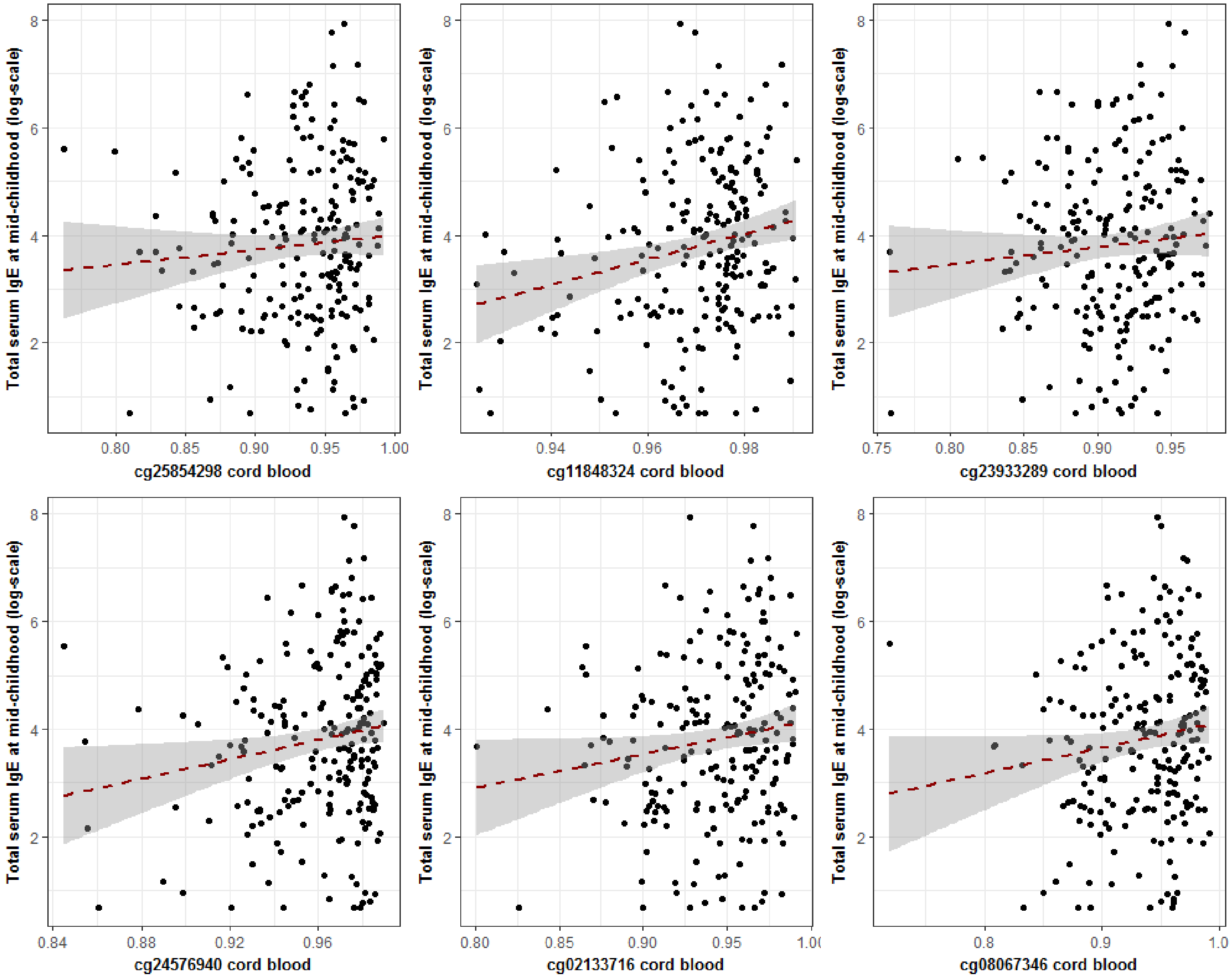

Figure S2. QQ plots.

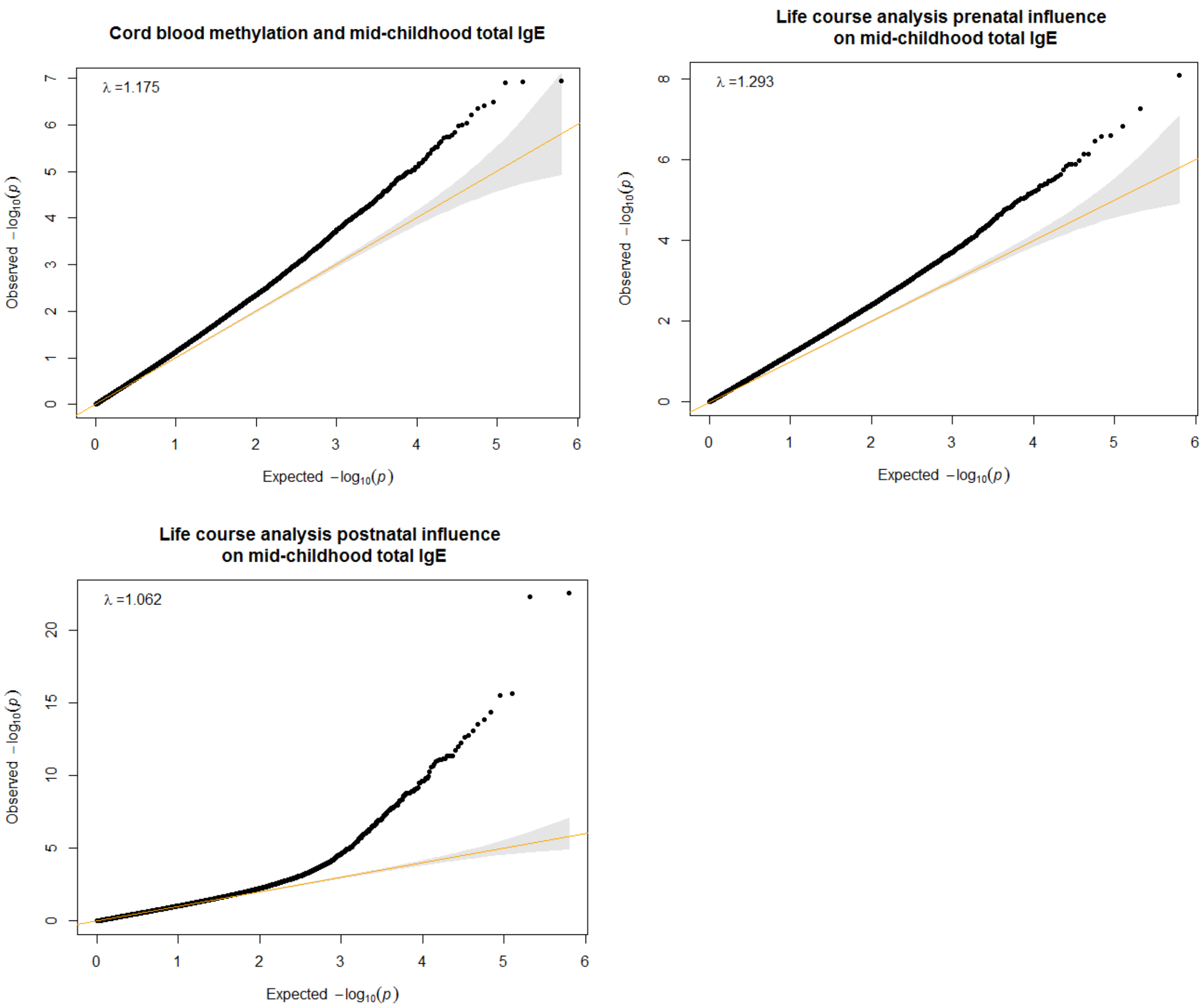

Figure S3a. Trend of top hits (reflect life course analysis—prenatal influence) (N=56).

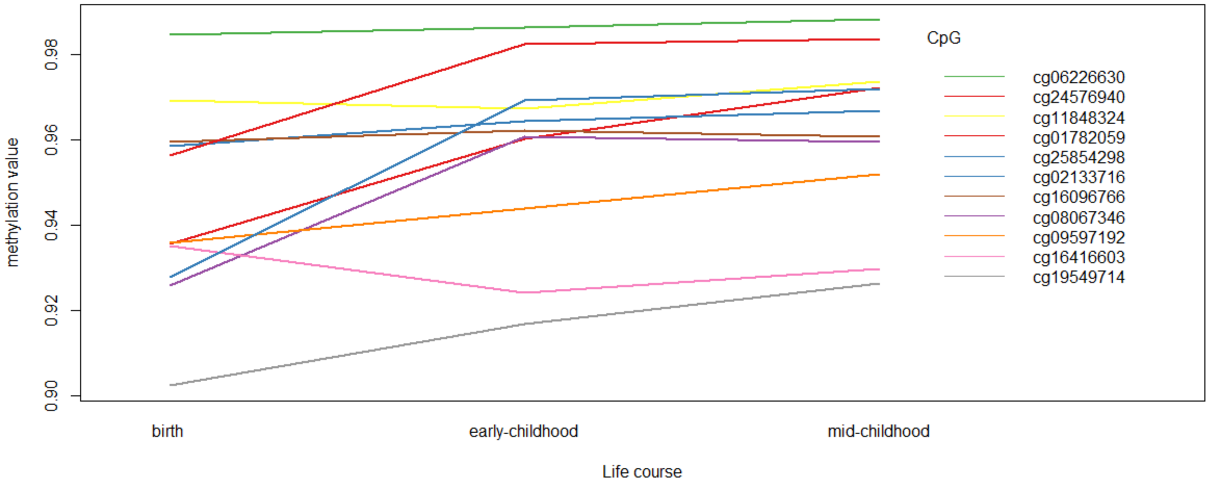

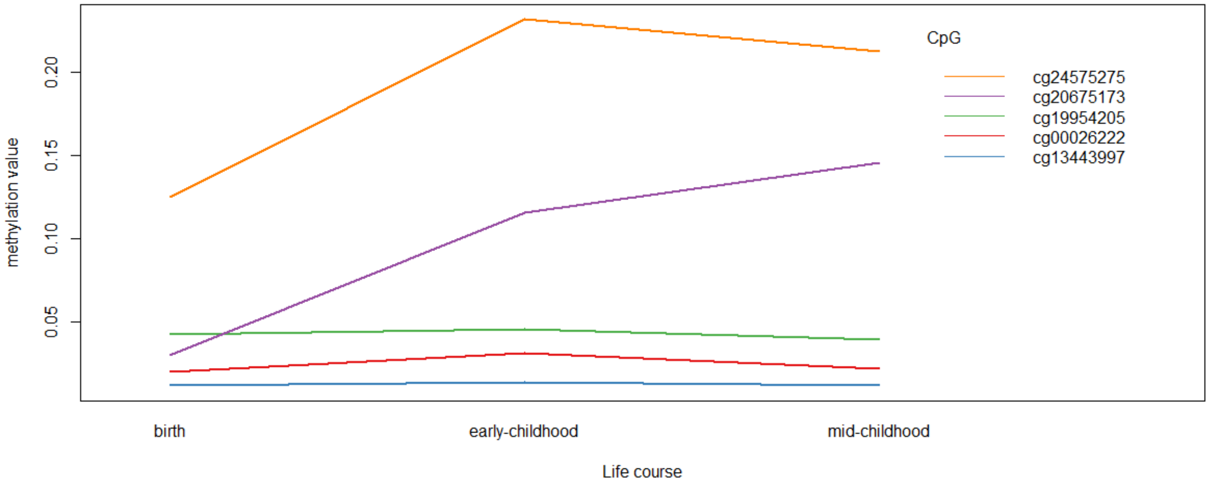

Figure S3b. Trend of top hits (reflect life course analysis—prenatal and postnatal influence) (N=56).

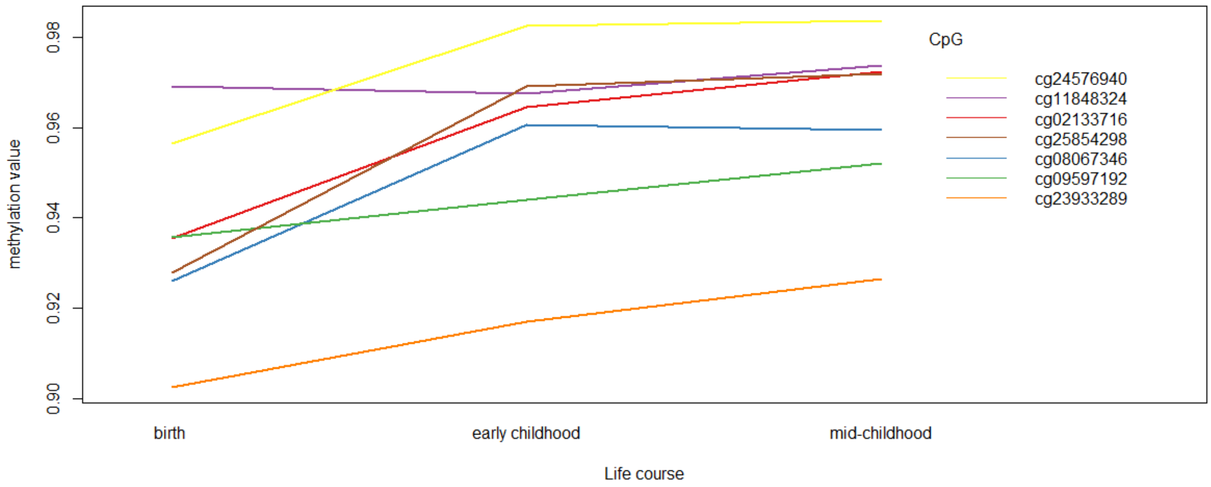

Figure S3c. Trend of top hits (reflect life course analysis—postnatal influence) (N=56).

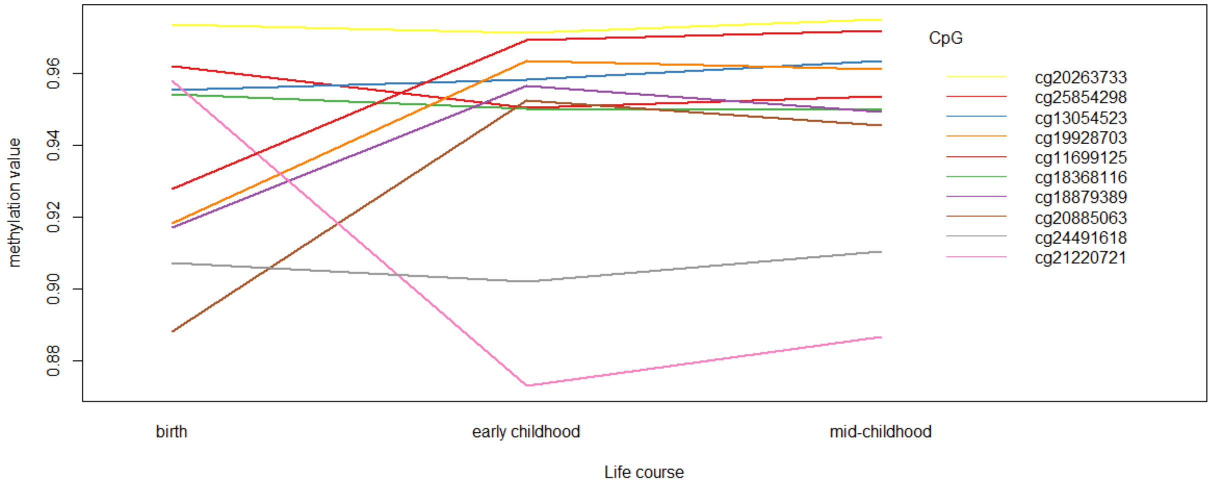

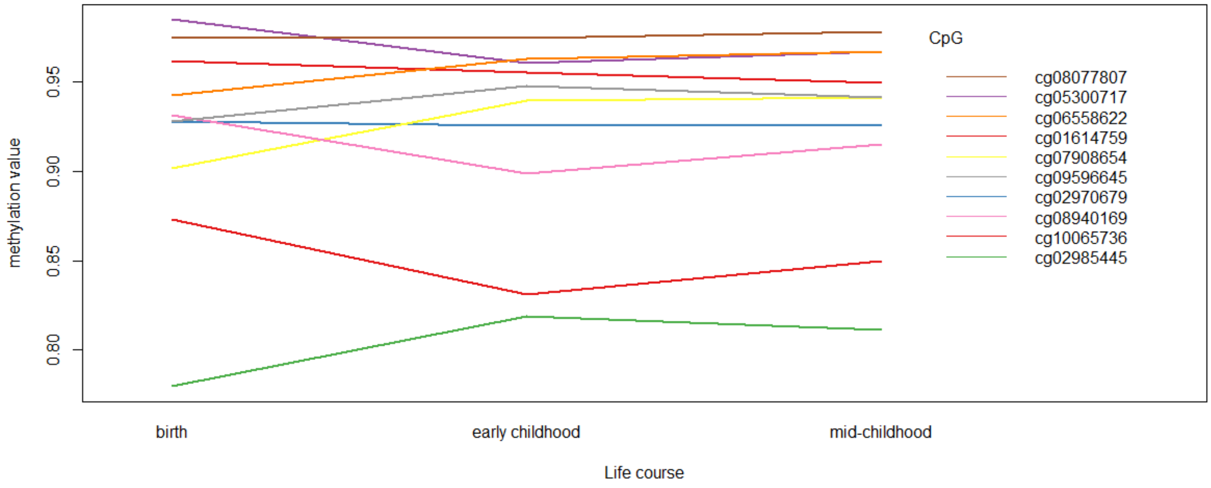

Figure S4. Distribution of the range of epigenome-wide significant methylation sites identified from the cord blood analysis and the life course analysis.

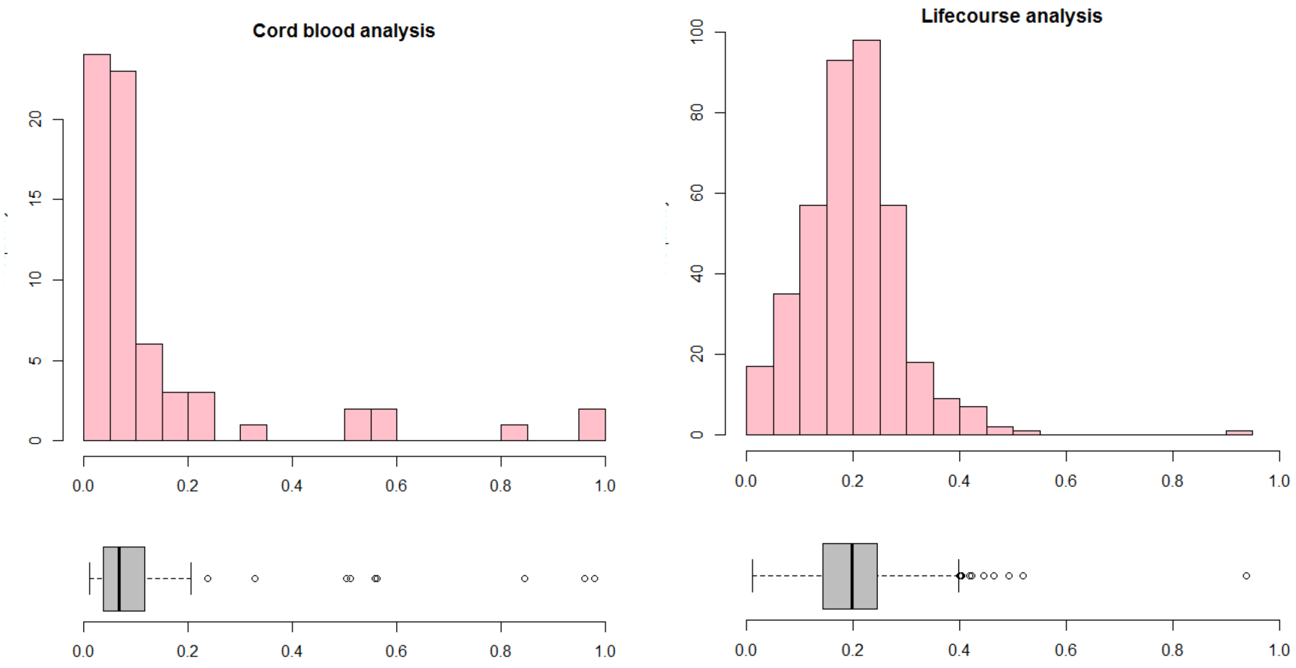

Figure S5. QQ plot of cord blood analysis using blood reference panel to adjust for cellular hetegeneity.

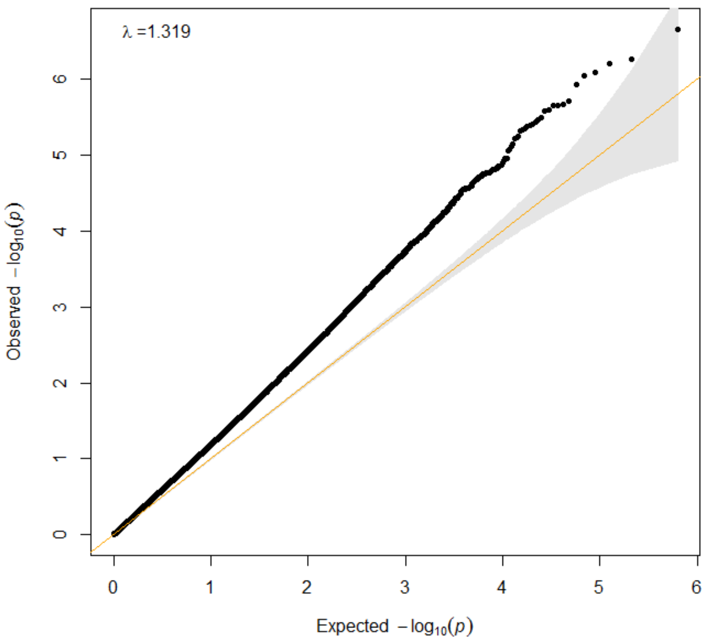

Supplement: Supplementary file 2 — Figure S1. Scatter plot (cord blood DNA methylation and mid-childhood total serum IgE). Scatter plot (life course analysis: prenatal influence—top 6 methylation sites). Scatter plot (life course analysis: postnatal influences—top 6 methylation sites). Scatter plot (life course analysis: postnatal influences (asthma pathway)—top 6 methylation sites). Scatter plot (life course analysis—contribution of prenatal and postnatal influences—top 6 methylation sites). Figure S2. QQ plots. Figure S3a. Trend of top hits (reflect life course analysis—prenatal influence) (N = 56). Figure S3b. Trend of top hits (reflect life course analysis—prenatal and postnatal influence) (N = 56). Figure S3c. Trend of top hits (reflect life course analysis—postnatal influence) (N = 56). Figure S4. Distribution of the range of epigenome-wide significant methylation sites identified from the cord blood analysis and the life course analysis. Figure S5. QQ plot of cord blood analysis using blood reference panel to adjust for cellular heterogeneity. (PDF 330 kb) [file 13148_2018_488_MOESM2_ESM.pdf]
